# Comparison of neuromuscular development in two dinophilid species (Annelida) suggests progenetic origin of *Dinophilus gyrociliatus*

**DOI:** 10.1186/s12983-016-0181-x

**Published:** 2016-11-08

**Authors:** Alexandra Kerbl, Elizaveta G. Fofanova, Tatiana D. Mayorova, Elena E. Voronezhskaya, Katrine Worsaae

**Affiliations:** 1 Marine Biological Section – Department of Biology, University of Copenhagen, Universitetsparken 4, 2100 Copenhagen, Denmark; 2 Laboratory of Developmental Neurobiology, Koltzov Institute of Developmental Biology RAS, 26 Vavilova Str., Moscow, Russia; 3 Laboratory of Neurobiology, National Institute of Neurological Disorders and Stroke, NIH, 49 Convent Dr., Bethesda, MD USA

**Keywords:** Meiofauna evolution, Paedomorphosis, Sexual dimorphism, Sister species, Nervous system, Musculature, Ciliation, Interstitial

## Abstract

**Background:**

Several independent meiofaunal lineages are suggested to have originated through progenesis, however, morphological support for this heterochronous process is still lacking. Progenesis is defined as an arrest of somatic development (synchronously in various organ systems) due to early maturation, resulting in adults resembling larvae or juveniles of the ancestors. Accordingly, we established a detailed neuromuscular developmental atlas of two closely related Dinophilidae using immunohistochemistry and CLSM. This allows us to test for progenesis, questioning whether i) the adult smaller, dimorphic *Dinophilus gyrociliatus* resembles a younger developmental stage of the larger, monomorphic *D. taeniatus* and whether ii) dwarf males of *D. gyrociliatus* resemble an early developmental stage of *D. gyrociliatus* females.

**Results:**

Both species form longitudinal muscle bundles first, followed by circular muscles, creating a grid of body wall musculature, which is the densest in adult *D. taeniatus*, while the architecture in adult female *D. gyrociliatus* resembles that of prehatching *D. taeniatus*. Both species display a subepidermal ganglionated nervous system with an anterior dorsal brain and five longitudinal ventral nerve bundles with six sets of segmental commissures (associated with paired ganglia). Neural differentiation of *D. taeniatus* and female *D. gyrociliatus* commissures occurs before hatching: both species start out forming one transverse neurite bundle per segment, which are thereafter joined by additional thin bundles. Whereas *D. gyrociliatus* arrests its development at this stage, adult *D. taeniatus* condenses the thin commissures again into one thick commissural bundle per segment. Generally, *D. taeniatus* adults demonstrate a seemingly more organized (= segmental) pattern of serotonin-like and FMRFamide-like immunoreactive elements. The dwarf male of *D. gyrociliatus* displays a highly aberrant neuromuscular system, showing no close resemblance to any early developmental stage of female *Dinophilus*, although the onset of muscular development mirrors the early myogenesis in females.

**Conclusion:**

The apparent synchronous arrest of nervous and muscular development in adult female *D. gyrociliatus,* resembling the prehatching stage of *D. taeniatus,* suggests that *D. gyrociliatus* have originated through progenesis. The synchrony in arrest of three organ systems, which show opposing reduction and addition of elements, presents one of the morphologically best-argued cases of progenesis within Spiralia.

## Background

Meiofaunal life forms (specimens passing through a sieve with a mesh-size of 1 mm, while being retained on a sieve with 42 μm mesh-size, [1]) are represented in most extant macrofaunal bilaterian lineages as well as constituting numerous independent lineages (e.g., Acoela, Kinorhyncha, Gastrotricha, Gnathostomulida, etc. [2–4]). The meiofaunal lineages Gnathifera and Rouphozoa (with macroscopic forms of platyhelminths nested within) were recently shown to branch off first within Spiralia [3, 5]). As a consequence hereof, the ancestral spiralian condition might have been an acoelomate to pseudocoelomate, microscopic bodyplan with direct development (possibly inhabiting the interstitial realm) [3]. In Annelida, however, the most basally branching groups are macroscopic, therefore suggesting that meiofaunal groups such as the interstitial family Dinophilidae evolved by either gradual miniaturization or underdevelopment (paedomorphosis) [5, 6]. Paedomorphosis is caused by a change in developmental timing due to early offset (progenesis), late onset (post displacement) or slower developmental rate (neoteny). All these changes can be either local or global processes and result in the underdevelopment of either individual characters or sets of characters [7–21]. Global progenesis is considered a common pathway of the evolution of microscopic annelids from macroscopic juveniles, which grow up in the same interstitial environment between the sand grains. Progenesis hereby offers the possibility to become permanently small and colonize the favorable interstitial habitat through an inherited arrest of somatic growth in a larval or juvenile ancestor by a single speciation event, possibly initiated by an early maturation [5, 7–12, 14, 17, 22–29].

Dinophilidae has been discussed in early studies to represent ancestral features within Annelida, when it was considered an archiannelid lineage alongside other interstitial annelids, due to its’ members microscopic size and simple morphology [30–34]. It was later argued from morphological studies to have developed via progenesis from a primarily large ancestor because of its simple morphology and similarity to juveniles of macrofaunal families such as Dorvilleidae [10, 11, 14, 17, 22, 35–37]. The relationship of Dinophilidae to other annelids is still debated with a recent phylogenomic study [5], which is suggesting it to be part of the clade Orbiniida (with low support) together with the macrofaunal family Orbiniidae as well as the meiofaunal families Nerillidae, Parergodrilidae, Diurodrilidae and *Apharyngtus*. However, another study did not consider its position sufficiently supported [25], and none of the studies could determine the closest relative.

Dinophilids are 1 to 3 mm long, with all species counting 6 segments and lacking appendages, parapodia, and chaetae. They have externally indistinct segmentation, recognized only by the arrangement of transverse ciliary bands [32, 38, 39] and internal features such as lateral nerves, commissures, and nephridia [11, 40, 41]. The family Dinophilidae contains *Trilobodrilus* with six described species [30, 42–46] and *Dinophilus,* which is represented by approximately ten species [32, 38, 47–52], since the validity of several additional taxa is questioned due to ambiguous or insufficiently detailed morphological descriptions. Very few species have been barcoded, so further molecular sampling may reveal a higher cryptic diversity (Worsaae et al. unpublished). Two different morphotypes can be distinguished within *Dinophilus*: 1) monomorphic dinophilids with a long life cycle including an encystment stage for up to eight months [31, 53], 2) strongly dimorphic dinophilids with a rapid life cycle of only three weeks for adult females and less than a week for dwarf males [54, 55]. The dimorphic type has “normal-sized” females and miniature dwarf males [56–59], while in the monomorphic species the sexes cannot be distinguished from each other by outer morphological characters [56, 57]. Development in both morphotypes is direct, as found in most meiofaunal species, but different to the indirect life cycle of the annelid species used for developmental studies so far (e.g. *Capitella teleta* [60–62], *Platynereis sp.* [63, 64]). While the five to seven species of the monomorphic, bigger, orange type are limited to shallow colder waters of the arctic, subarctic and boreal coasts of e.g. Newfoundland, Greenland, Sweden, Denmark, Great Britain, and Russia [33, 38, 39, 48, 53], the hyaline, smaller, dimorphic type can be found in both boreal and temperate waters such as in Denmark [55, 58], France (pers. obs.), the Mediterranean [40], Brazil [65], North Carolina [66] and China [67]. Both monomorphic and dimorphic species of *Dinophilus* are found in the intertidal and subtidal region, where they are grazing on biofilm and small algae overgrowing macroalgae or in the interstices among sand grains in shallow waters [30, 34, 54, 68].

Despite several anatomical studies [11, 32, 40, 41, 56, 57, 69–72], little is known about the neuromuscular development in *Dinophilus*, which is thoroughly assessed in this study. However, previous studies did already assess the adult stages, stating that the musculature consists mainly of the pharyngeal [73] and body wall musculature, which is specified as layers of circular, diagonal, and longitudinal musculature [39]. The nervous system has likewise been investigated in mainly adults, assessing the relatively simple brain and a ventral nervous system consisting of five to seven longitudinal nerve cords [11, 40, 41, 71]. These are connected by one (in monomorphic) or three commissures (in dimorphic species) per segment, respectively. The neuromuscular system in *D. gyrociliatus* dwarf males is altered significantly from the pattern seen in females and also in other dinophilid males [56, 57]. Based on their diminutive size and ciliary pattern, they have been proposed to resemble a trochophore larva [56, 57], the resemblances however seem to be superficial.

Due to the size and morphological differences between the two morphotypes, it is proposed in this study that the smaller and simpler built *D. gyrociliatus* Schmidt, 1857 as representative of the dimorphic, fast developing morphotype has originated through a second progenetic process from the possibly already paedomorphic ancestor of Dinophilidae, which was most likely resembling the more complex and larger forms found in *D. taeniatus*. The evolutionary unravelling of *D. gyrociliatus* is further complicated by their possession of dwarf males, since males of *D. gyrociliatus*-ancestors probably have undergone a separate or ‘third’ progenesis relative to the females [58, 74], while *D. taeniatus* Harmer 1889 as representative of the monomorphic group with prolonged life cycle as well as the related dinophilid taxon, *Trilobodrilus*, have “normal-sized” males [31, 53, 75].

We hereby aim to establish a reference model for direct developing meiofaunal annelids by examining the neuromuscular system and its development in both sexes of *D. gyrociliatus* and *D. taeniatus* with immunohistochemistry and confocal laser scanning microscopy (CLSM), thereby also facilitating comparison across species and sexes. We will further examine whether the seemingly simpler morphology in female *D. gyrociliatus* reflects earlier developmental stages of *D. taeniatus* and whether the dwarf males resembles even earlier developmental stages of females, hereby seeking support for the hypotheses on a progenetic origin of the male and female *D. gyrociliatus*.

## Methods

### Specimens

Two different populations of *Dinophilus gyrociliatus* (originally from Xiamen, China and Naples, Italy) and two different populations of *D. taeniatus* (collected at the White Sea, Russia and in Quequertarsuaq, Disko Island, Greenland) were examined in the present study. No significant morphological intraspecific variations were detected between the populations. The presented illustrations are mainly based on *D. gyrociliatus* from lab cultures originally from China and *D. taeniatus* collected at the White Sea, Russia.

#### Dinophilus gyrociliatus

One culture of *D. gyrociliatus* was established by Bertil Åkesson at University of Gothenburg in the 1980’s from specimens sampled in Xiamen, China. A subsample of this culture is now kept at the Marine Biological Section, University of Copenhagen, Denmark, where the animals are maintained in seawater (salinity 28‰) at 18 °C and fed spinach twice a month after exchanging the water. Another culture of *D. gyrociliatus* (originally sampled in Naples, Italy) is kept in the institute of Developmental Biology RAS, Moscow, Russia. The worms are cultured in artificial seawater with 33‰ salinity at 20 °C and fed nettle once a week after exchanging the water.

For establishing the life cycle and stage-specific sampling, some females were separated from the main culture and checked on a daily basis. Newly laid cocoons were transferred to dishes, tracked and fixed after two days and subsequently every 12 h until hatching (after six days) for the establishment of the developmental series.

#### Dinophilus taeniatus

The Greenlandic specimens of *Dinophilus taeniatus* were obtained during a field trip to Disko Island, Southwest Greenland, from the shallow waters in the intertidal region in Quequertarsuaq harbour. The Russian specimens of *D. taeniatus* were obtained at the Pertsov White Sea Biological Station (White Sea, Russia). The worms were collected during low tide at the upper sublittoral zone. The culture of *D. taeniatus* was reared in the laboratory in natural filtered seawater at 10 °C and was checked twice a day for the presence of cocoons.

The cocoons were transferred to separate Petri dishes and kept in filtered seawater until fixation after four days and then every 24 h until hatching (approximately after 21 days). Juvenile and adult stages were also fixed similar to *D. gyrociliatus*.

Embryonic development is characterized by different duration of respective stages. We therefore use morphological markers (internal and external ciliary structures such as ciliary bands and ventral ciliary field, musculature and nervous system) and the sequence of their formation to compare the stages of the neuromuscular system in both morphotypes.

#### Staging of dinophilid development

We categorized *Dinophilus* development into 5 stages: early embryo (2.5–3 days after cocoon deposition in *D. gyrociliatus* and 5–6 days after cocoon deposition in *D. taeniatus*), late embryo (several ciliary bands and the ventral ciliary field developed, 4.5 days in *D. gyrociliatus* and 10–14 days in *D. taeniatus*,), prehatching/hatching (just before hatching from the fertilization envelope and – later on – the cocoon, 5.5–6 days in *D. gyrociliatus* and 14–21 days in *D. taeniatus*), juvenile (6.5–12 days in *D. gyrociliatus* and 21–40 days in *D. taeniatus*) and adult (12 and more days in *D. gyrociliatus* and 40 and more days in *D. taeniatus*,). The morphology is described in detail for *D. gyrociliatus* females and description of *D. taeniatus* is mainly focused on differences and similarities.

### Immunohistochemistry and confocal laser scanning microscopy (CLSM)

Specimens (at least ten specimens per stage and used antibody) were anesthetized with isotonic MgCl_2_ prior to fixation with 3.7 % paraformaldehyde in phosphate buffered saline (PBS, pH 7.4) at room temperature (RT); embryos were manually extracted from the cocoon and the fertilization envelope prior to fixation. Double as well as quadruple stainings were applied to investigate characters in the muscular, nervous, and ciliary system. These stainings included F-actin staining (Alexa Fluor 488-labelled phalloidin, A12379, INVITROGEN, Carlsbad, USA), DNA-staining (405 nm fluorescent DAPI, included in the embedding medium Vectashield) and immunostaining (monoclonal mouse anti-acetylated α-tubulin (T6793, SIGMA, St. Louis, USA), polyclonal anti-mouse anti-tyrosinated tubulin (T9028, SIGMA), polyclonal rabbit anti-serotonin (5-HT, S5545, SIGMA) and anti-FMRFamide (20091, IMMUNOSTAR, Hudson, USA)). Prior to adding the primary antibody-mix, the samples were preincubated with 1 % PBT (PBS + 1 % Triton-X, 0.05 % NaN_3_, 0.25 % BSA, and 5 % sucrose). Afterwards, samples were incubated for up to 24 h at RT in the primary antibodies mixed 1:1 (in a final concentration of 1:400). Subsequently, following several rinses in PBS and 0.1 % PBT, specimens were incubated with the appropriate secondary antibodies conjugated with fluorophores (also mixed 1:1, in a final concentration of 1:400, goat anti-mouse labelled with CY5 (115-175-062, JACKSON IMMUNO-RESEARCH, West Grove, USA), goat anti-rabbit labelled with TRITC (T5268, SIGMA)) for up to 48 h at RT. This step was followed by incubation for 60 min in Alexa Fluor 488-labeled phalloidin solution (0.33 M phalloidin in 0.1 % PBT) after and prior to several rinses in PBS. Thereafter, specimens were mounted in Vectashield (including DAPI, VECTOR LABORATORIES, Burlingame, USA). The prepared slides were examined using an OLYMPUS IX 81 inverted microscope with a Fluoview FV-1000 confocal unit at the Marine Biology Section of the University of Copenhagen (property of K. Worsaae) and a Nikon A1 CLSM at the White Sea Biological Station. Acquired z-stacks were exported to the IMARIS 7.0 (BITPLANE SCIENTIFIC SOFTWARE, Zürich, Switzerland) software package to conduct further three-dimensional investigations and prepare representative images.

### Image processing

Brightness, saturation, and contrast were adjusted in Adobe Photoshop CC 2015 (ADOBE Systems Inc., San Jose, USA) prior to assembling figure plates in Adobe Illustrator CC 2015, where also schematic drawings were created.

## Results

### Overall morphology and life cycle

#### *Dinophilus gyrociliatus* females and dwarf males

**Fig. 1:**
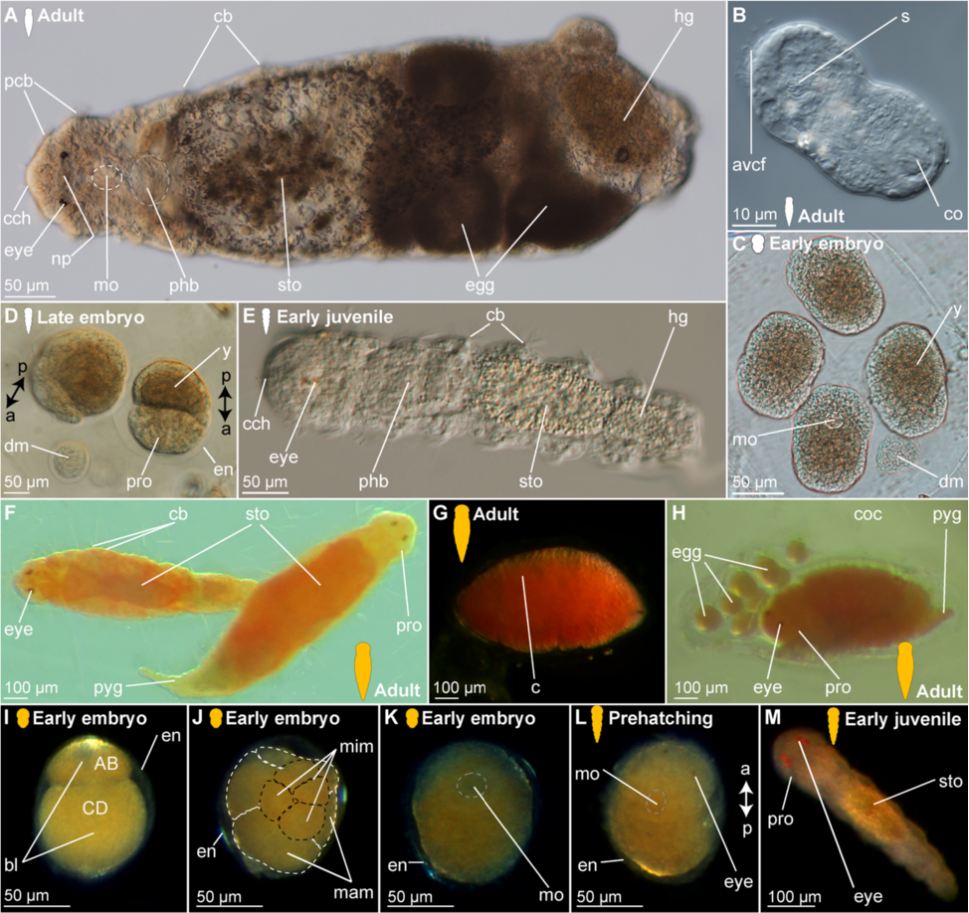
Light microscopic pictures of different life stages of *Dinophilus gyrociliatus* and *D. taeniatus*. Stages are indicated by silhouettes (*D. gyrociliatus* in white and *D. taeniatus* in orange), and the assignment to the respective stage next to them. Double-arrows indicate the antero-posterior axis (a-p) in the animals at prehatching stage. **a**-**e** *Dinophilus gyrociliatus*, **a** adult female, dorsal view, **b** dorsal view of an adult dwarf male, **c** cocoon with female embryos and dwarf males at 2 days after deposition, **d** cocoon with females and one dwarf male at 5 days after deposition (prehatching embryos), **e** early juvenile female, dorsolateral view, **f**-**h** *D. taeniatus*, **f** copulating male (on the left side) and female (on the right side), dorsal view, **g** encysted worm, **h** female next to a cocoon with eight eggs in dorso-lateral view, **i**-**l** embryogenesis, **i** two blastomere-stage with the apical pole up, **j** morula stage, **k** postgastrulation stage in ventral view, **l** prehatching embryo curling inside fertilization envelope with its anterior end up, **m** juvenile in dorsal view. Abbreviations: avcf – anteroventral ciliary field, bl – blastomere, c – cyst, cb - ciliary band, cch – compound cilia of the head, co – copulatory organ, coc – cocoon, dm – dwarf male, en – fertilization envelope, hg – hindgut, mam – macromere, mim – micromere, mo – mouth opening, np – neuropil, pcb – prostomial ciliary bands, phb – pharyngeal bulb, pro - prostomium, pyg – pygidium, s – sperm, sto – stomach, y – yolk

**Fig. 2:**
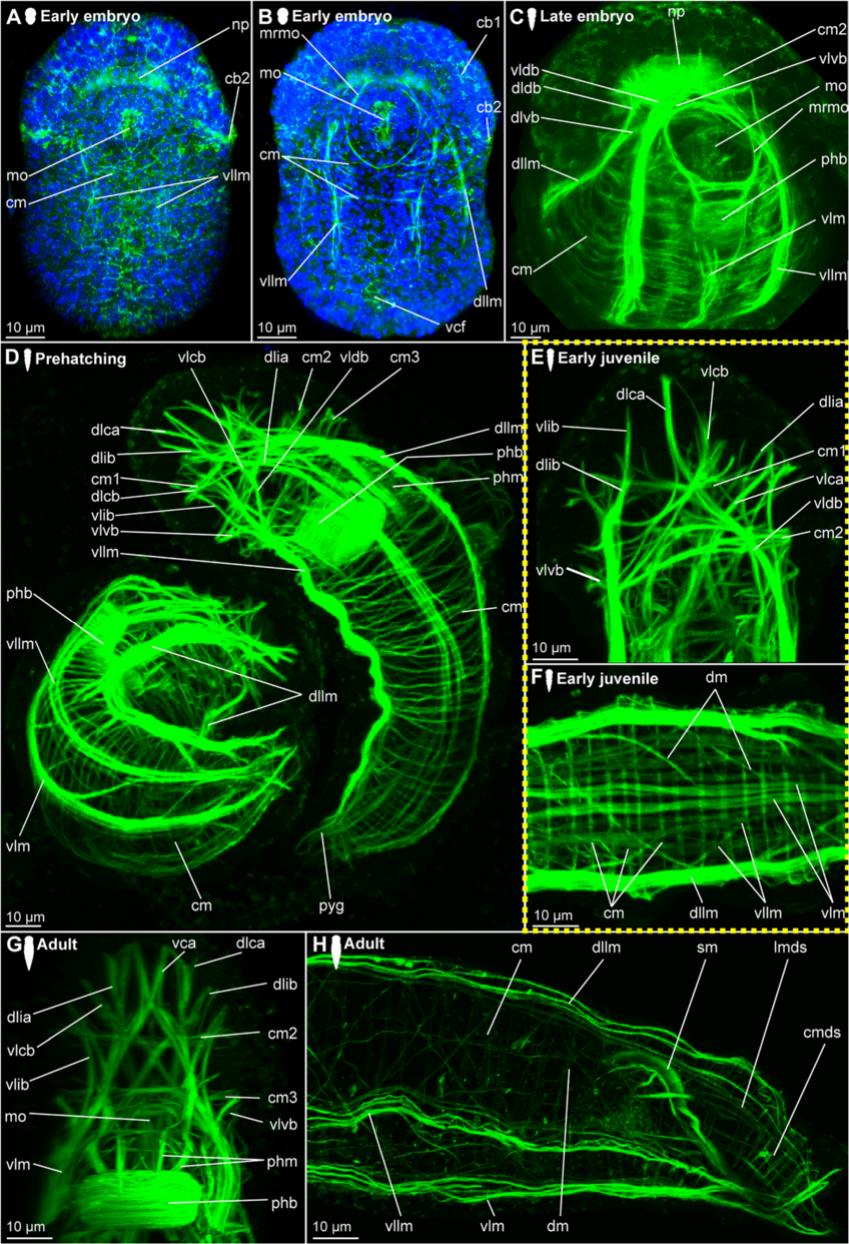
Myogenesis in *Dinophilus gyrociliatus* females. Phalloidin-labelled actin-filaments shown in green, labelling of DNA with DAPI shown in blue, animals are oriented with the anterior end up (**a**-**e**, **g**, **h**) or to the left (**f**). Stages are indicated by silhouettes next to the figure capture, and the assignment to the respective stage next to them. The first signs of difference between the two species *D. gyrociliatus* and *D. taeniatus* are emphasized by a yellow dashed-lined frame around the picture. **a** Ventral view of the onset of myogenesis in the early embryo (3 days after the egg is deposited), **b** Ventral view of the female *D. gyrociliatus* with ventrolateral and dorsolateral longitudinal muscles developed in the early embryo (3.5–4 days after the egg is deposited), **c** ventral view of the exogastrically curled females in the late embryo (5–5.5 days after the egg is deposited), **d** prehatching females (left female still curled exogastrically inside the egg layer, right female free inside the cocoon with the dorsolateral side up, 5.5–6 days after the egg is deposited), **e** dorsal view of the head musculature in an early juvenile female, **f** dorsoventral view of the trunk musculature with longitudinal, circular and diagonal elements in an early juvenile female, **g** ventral view of the head musculature in an adult female, **h** lateral view of the posterior region of an adult female. Abbreviations: cb1-2 –ciliary band 1–2, cm – circular muscle, cmds – circular muscle of the digestive system, dlca – contralatero-anterior branch of the dorsolateral longitudinal muscle, dlcb – contralateral branch of the dorsolateral longitudinal muscle, dldb – dorsal branch of the dorsolateral longitudinal muscle, dlia – ipsilatero-anterior branch of the dorsolateral longitudinal muscle, dlib – ipsilateral branch of the dorsolateral longitudinal muscle, dllm – dorsolateral longitudinal muscle, dlvb – ventral branch of the dorsolateral longitudinal muscle, dm – diagonal muscle, lmds – longitudinal muscle of the digestive system, mo – mouth opening, mrmo – muscular ring around the mouth opening, np – neuropil, phb – pharyngeal bulb, phm – pharyngeal muscle, pyg – pygidium, sm – sigmoid muscle, vca - contralatero-anterior branch of the ventral longitudinal muscle, vcf – ventral ciliary field, vlcb – contralateral branch of the ventrolateral longitudinal muscle, vldb – contralatero-dorsal branch of the ventrolateral longitudinal muscle, vldb – dorsal branch of the ventrolateral longitudinal muscle, vlib – ipsilatero-anterior branch of the ventrolateral longitudinal muscle, vllm – ventrolateral longitudinal muscle, vlm – ventral longitudinal muscle, vlvb – ventral branch of the ventrolateral longitudinal muscle

**Fig. 3:**
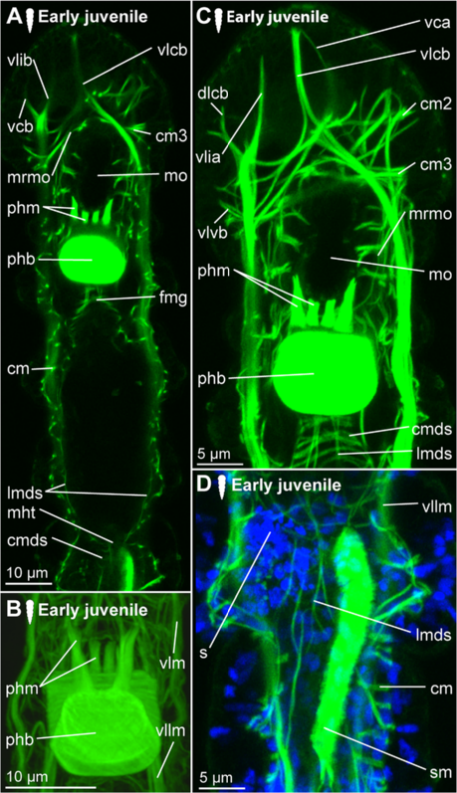
Musculature of the digestive system in juvenile *Dinophilus gyrociliatus* females. Phalloidin-labelled actin-filaments shown in green, labelling of DNA with DAPI shown in blue, animals are oriented with the anterior end up. Stages are indicated by silhouettes next to the figure capture, and the assignment to the respective stage next to them. **a** horizontal section through a juvenile female at the level of the sigmoid muscle, **b** detail of the pharyngeal bulb in dorsal view, **c** dorsal view of the head and pharyngeal musculature, **d** dorsal view of the posterior part of the body with sigmoid muscle and injected sperm lateral in an early juvenile female. Abbreviations: an – anus, cm – circular muscle, cmds – circular muscle of the digestive system, dlcb – contralateral branch of the dorsolateral longitudinal muscle, fmg – foregut-midgut transition, hg – hindgut, lmds – longitudinal muscle of the digestive system, mht – midgut-hindgut transition, mo – mouth opening, mrmo – muscular ring around the mouth opening, phb – pharyngeal bulb, phm – pharyngeal muscle, s – sperm, sm – sigmoid muscle, vcb – contralateral dorsal branch of the ventral longitudinal muscle, vlcb – contralateral branch of the ventrolateral longitudinal muscle, vlcb – contralatero-dorsal branch of the ventrolateral longitudinal muscle, vlia – anterior ipsilaterial branch of the ventrolateral longitudinal muscle, vlib – ipsilatero-anterior branch of the ventrolateral longitudinal muscle, vllm – ventrolateral longitudinal muscle, vlvb – ventral branch of the ventrolateral longitudinal muscle

**Fig. 4:**
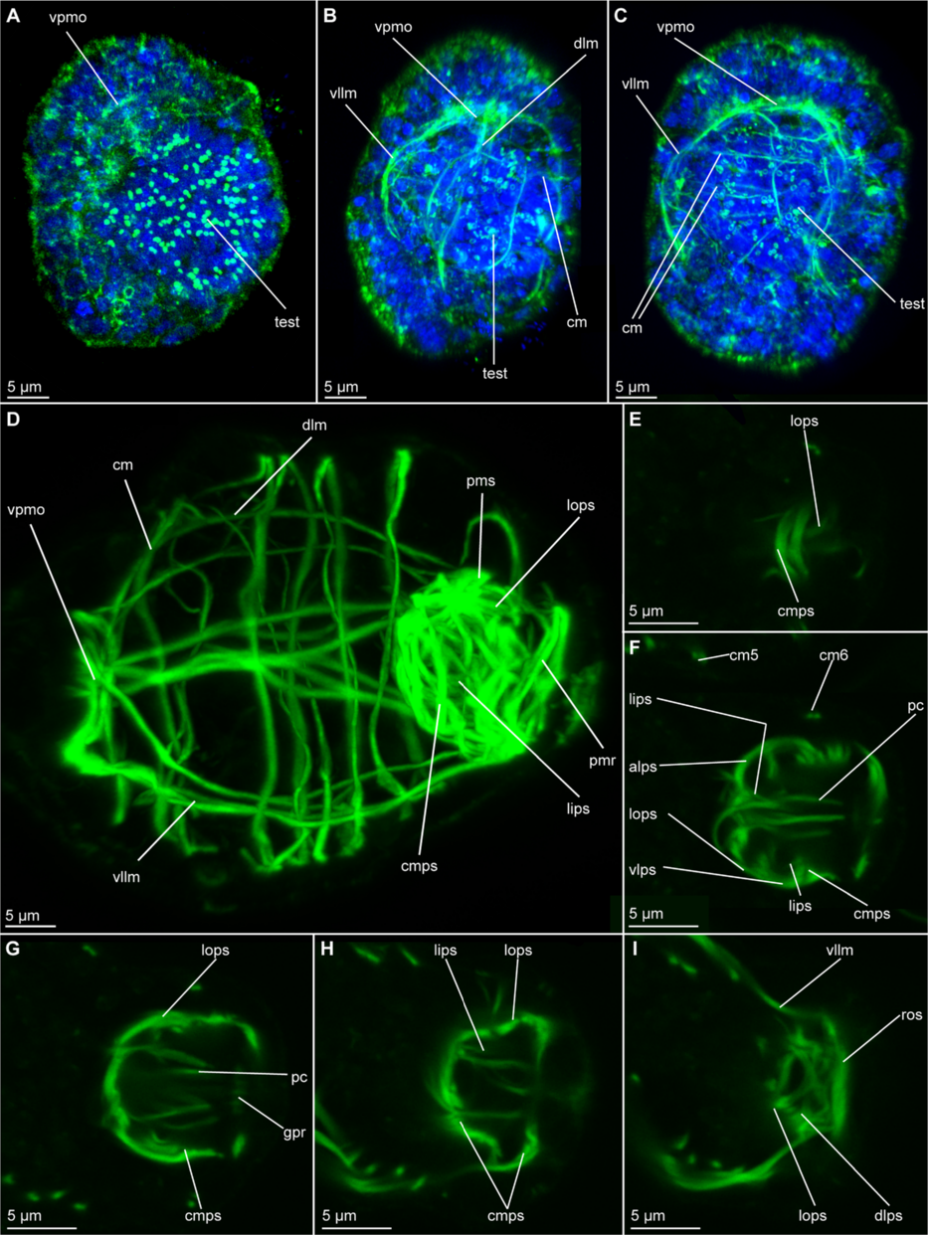
Muscular development in dwarf males of *Dinophilus gyrociliatus*. Phalloidin-labelled actin-filaments shown in green, labelling of DNA with DAPI shown in blue, animals are oriented with the anterior end up in dorso-ventral view (A-C), or to the left and in lateral view (D-I). **a** dorsal view of an one day old dwarf male with the anterior point of muscular origin formed; **b** dorsal region of the longitudinal and circular fibres forming in a two day old dwarf male in dorsal view; **c** ventral region of longitudinal and circular fibres in a two day old dwarf male, **d** musculature in a prehatching dwarf male; **e**-**i** section series with anterior to the left and posterior to the right through the copulatory organ in an adult dwarf male from the ventral (**e**) to the dorsal side (**i**), illustrating the penile cone (**f**, **g**) and the penile sheath (**e**-**i**). Abbreviations: alps – anterior loop of the outer sheath. cm – circular muscle, cmps – circular muscles of the penile sheath, dlm – dorsal longitudinal musculature, dlps – dorsal loop of the penile sheath, gpr – gonopore opening, lips – longitudinal inner muscles of the penile sheath, lops – longitudinal outer muscles of the penile sheath, pc – penile cone, pmr – posterior muscular ring, psm – penile sheath musculature, ros – ring around the gonopore formed by the outher sheath, test – testis, vllm – ventrolateral longitudinal muscle, vlps – ventral loop of the penile sheath, vpmo – ventroanterior point of muscular origin

The adult female’s body is cigar-shaped, ranges in length between 1.0 to 1.5 mm and has a diameter of approximately 75–150 μm (Fig. 1a). The body is very hyaline and therefore internal organs such as the digestive system with the prominent pharyngeal bulb as well as developing eggs can be seen (Fig. 1a). One transverse ciliary band is found per segment in this species (cb, Fig. 1a). Adult dwarf males are about 50 μm in length and 20 μm in width with a roughly elongated ovoid shape (Fig. 1b). They do not form a digestive system, but the penile region with the muscular copulatory organ is prominently developed (co, Fig. 1b). The deposited cocoons contain several big female and small male eggs (dm labelling the male egg developing into a dwarf male, Fig. 1c), which are present in an average ratio of 1 male (dm, Fig. 1c, d) to 2–4 female eggs ([46], Fig. 1c, d), with the cocoons containing at least one male and one female egg. Male and female eggs retain their size difference throughout development.

In contrast to the females, which were observed to hatch from the eggs after six days and mature afterwards, the dwarf males are already mature when hatching from the fertilization envelope (approximately five days after the cocoon has been deposited and before females hatch) and die one or two days later, after they fertilize the females inside the same cocoon and females passing in close proximity to the opened cocoon. Hereby the male has been observed to penetrate the body wall of the female and transfer sperm underneath the epidermis of the female in the posterior body region, where it is stored until the latter have developed eggs [76]. Hatching females and early juveniles are elongated and thin, with the individual ciliary bands located on broader body regions and thereby showing serial arranged structures (e.g. ciliary bands and intersegmental furrows) that suggest segmental arrangement of organ systems (Fig. 1e), while this pattern is continuously obscured when juveniles start feeding and extend their body circumference. The transition between juveniles and mature animals is fluent, with adults carrying eggs and dilating the posterior body region in the process (Fig. 1a).

The entire life cycle of the females from the time the cocoon is deposited to the time when the adult females lay their own cocoons takes a maximum of three weeks, including one week of embryonic development inside the cocoon (Fig. 1c-e).

#### *Dinophilus taeniatus* females and males

The external morphology in both males and females is similar to the one of *D. gyrociliatus* females described above, though the animals are larger (body length 2.3–3.1 mm, body width 100–300 μm, Fig. 1f). In contrast to *D. gyrociliatus*, the body of this species is strongly pigmented (animals are bright orange, Fig. 1f-m), differences between the sexes cannot be defined by outer morphology at any stage, except when females are carrying eggs. In contrast to *D. gyrociliatus*, where the dwarf male mainly fertilizes (pre-)hatching females of the same cocoon, copulation in *D. taeniatus* occurs in adults after hatching. When copulating (Fig. 1f), the male penetrates the body wall of the female with the penis. After a certain period of time, when encystment may take place (Fig. 1g), the female deposits a cocoon with eggs of both sexes, which cannot be distinguished by neither size, nor organization, nor colouration (Fig. 1h). Embryonic development takes two to three weeks; cleavage starts right after oviposition. *Dinophilus* is in general characterized by unequal, holoblastic spiral cleavage (Fig. 1i) resulting in a morula stage (Fig. 1j). The mouth opening is formed during gastrulation (mo, Fig. 1k). The embryos elongate and curl up inside their fertilization envelope at prehatching stage with their ventral side facing the fertilization envelope (Fig. 1l). The developmental sequence (i.e. the sequence of formation of musculature, ciliary structures and nerves) resembles that of female *D. gyrociliatus*, and thereby enables comparisons between specific stages. Similar to *D. gyrociliatus* (1A, E), the juvenile leaving the fertilization envelope resembles the adult (Fig. 1f, m).

### Musculature

#### *Dinophilus gyrociliatus* females

##### Embryonic development

*Body wall musculature.* The first signs of muscular development can be detected after gastrulation (approximately 1.5–2 days after cocoon deposition), when a pair of ventrolateral longitudinal muscles (vllm) forms posterior to the mouth opening and then extends towards the anterior and the posterior end of the body (Fig. 2a, b). Subsequently, additional fibres join these, and a dorsolateral pair of longitudinal muscles (dllm, Fig. 2b) is formed, as well as a muscular ring around the mouth opening (mrmo, Fig. 2 b). Prior to elongation and curling of the animal inside the fertilization envelope, a pair of ventral longitudinal muscle bundles (vlm) is developed (Fig. 2c). They move medially and converged along the midline, embracing both the mouth opening (mo) and the ventral side of the developing pharyngeal bulb (phb, Fig. 2c). All longitudinal muscle bundles extend anteriorly into the prostomium, where they ramify towards the periphery, though their exact paths cannot be unravelled in early stages (Fig. 2c).

First fragments of circular muscles (cm) start forming at the ventral side external to the longitudinal muscles at the same time as the ventrolateral and dorsolateral muscle bundles can be detected (Fig. 2a, b). Though several circular muscles are now added from anterior to posterior, they are incomplete in the earlier developmental stages (Fig. 2a-c), extending from the ventral towards the dorsal side, where they finally fuse at prehatching stage (Fig. 2d). In the late embryo stage, the circular muscles are forming an almost continuous sheath (Fig. 2c), which is not retained in later stages as the distance between the circular muscles increases (Fig. 2d-g).

*Prostomial musculature.* At the onset of muscular development, no muscles are formed anterior to the mouth opening. The developing brain and it neuropil, however, seem to be labelled by phalloidin, too, which is probably reacting to neuronal f-actin as was already shown previously in a wide range of animals such as molluscs [77, 78] and crustaceans [79] (Fig. 2a-c), and therby not related to musculature. Later on, the longitudinal muscle bundles of the posterior part of the body extend anteriorly (Fig. 2c), where they ramify and are joined by muscles emerging from the muscular ring around the mouth opening (mrmo, Fig. 2c). Supplementing these ramifications of the longitudinal muscles, three circular muscles are formed in the developing prostomium, which can be detected external to the attachment sites of the branching longitudinal muscles (cm1-3, Fig. 2c, d). During earlier developmental stages, the musculature is mainly dorsal to the neuropil (Fig. 2c), but also extends ventrally around the brain during subsequent stages (Fig. 2d, e, g, h). A more complete assessment of the pattern is possible in the hatching and juvenile stages (see below).

*Musculature of the digestive system.* The pharyngeal bulb (phb) is the most prominent and first developed part of the musculature of the digestive system emerging rather late in embryogenesis (approximately four days after cocoon deposition, Fig. 2c). The pharyngeal bulb itself consists of a tightly arranged stack of 27 plate-shaped muscle cells and dorsal and ventral longitudinal muscles [72], and is located posterior to the mouth opening (Figs. 2d).

The pharyngeal region differentiates in the developing embryo (Fig. 2b), and though cellular changes can be observed starting with the invagination of the mouth, muscular details can be detected much later. Similarly, the gut shows cellular differentiation of the adjacent cells prior to the formation of longitudinal and circular muscles, which can be detected after the formation of the pharyngeal bulb (4.5–5 days after the eggs have been deposited, Fig. 2d). However, the denser muscular layer of the body wall complicates the identification of the thin musculature of the alimentary channel. Compared to the longitudinal and circular muscles of the body wall, the respective elements in the digestive system were observed represented by one or two fibres only and spaced further apart.

##### Hatching & early juvenile stages

*Body wall musculature.* The layout of the longitudinal and circular muscles does not change significantly from the pattern detected during embryonic development, since only the dorsolateral longitudinal muscle bundles (dllm, Fig. 2d, f), which have been (ventro-)lateral in earlier stages, are shifted to the dorsal side. Internal to the longitudinal muscles, diagonal muscles (dm) are formed, which wind spiral-like around the body, starting at the level of the mouth opening and extending towards the posterior end of the animal (Fig. 2f). Their pattern does not seem to be fixed in development, since the muscles are arranged parallel to each other in some animals without any chiasmata, while the fibres are crossing each others’ paths more regularly in others.

*Prostomial musculature.* The musculature in the prostomium gets more defined, with additional fibres extending from the longitudinal muscle bundles on the ventral side of the body more dorsally, but also extending from the pharyngeal bulb to both the ventral and the dorsal side of the body (Figs. 2d, e, 3a, c). The circular muscles of the prostomium, in contrast to those of the body, consist of several fibres (two to seven, Figs. 2d, e, 3a, c). The ventrolateral longitudinal muscle bundles (vllm) extend ventrolaterally in a straight line to the level of the third circular muscular ring, where they then split into several branches of different thickness: The thinnest strand consists of one to a maximum of three fibres and extends ventrally to the prostomial epidermis anterior to the second circular muscle band (vlib, Figs. 2d, e, 3a). An additional muscle strand extends to the anterior tip ipsilateral to the midline (vlia, Fig. 3c). Furthermore, one strand extends contralaterally and connects to the epidermis at the level of the first circular muscle (vlca, Fig. 2e), and a short strand is directed more posterior and to the ventral side (vlvb, Fig. 2d, e).

The paths of the ventral and dorsolateral muscle bundles are less complex, but also show one to two splits: the dorsolateral muscle bundle bifurcates already anterior to the pharyngeal bulb into two strands of similar thickness, which are extending to the dorsolateral and ventrolateral side of the prostomium to the level of the first circular muscle. While one bundle is crossing the midline of the body and remains dorsolateral, extending contralaterally to the anterior tip (dlca, Fig. 2d, e), the lateral proportion extends ipsilateral to the first circular muscle (dlia, Fig. 2d, e). The third bundle is located most medial and extends contralaterally to the third circular muscle (dlcb, Figs. 2d, 3c). The ventral muscle bundles also split and traverse from the ventral to the dorsal side, also forming two furcations at this stage: a contralateral bundle extending to the epidermis at the level of the first circular muscle (vca, Fig. 3c) and another contralateral bundle extending to the level posterior to the third circular muscle (vcb, Fig. 3a). This pattern, once developed, can also be found in adult specimens of *D. gyrociliatus* female (Fig. 2g), though they get more refined and further splits are added.

*Musculature of the digestive system.* Additional circular fibres (cmds) form external to the longitudinal muscles of the gut musculature (lmds), extending from the mouth opening to the dorsal anus (Fig. 3a, c, d). Besides the muscular pharyngeal bulb, only a thin muscular ring is formed around the mouth opening (mrmo, Fig. 3a, c) and several thin circular fibres (spaced closer together than in the stomach (= midgut) and hindgut) are detected in the foregut (Fig. 3c). A thin muscular ring is formed around the anus (Fig. 2h). Additionally, an unpaired muscle traces the hindgut from the midgut-hindgut-transition to the ventral side anterior to the anus (sigmoid muscle – sm, Figs. 2h, 3d).

##### Adult

**Fig. 5:**
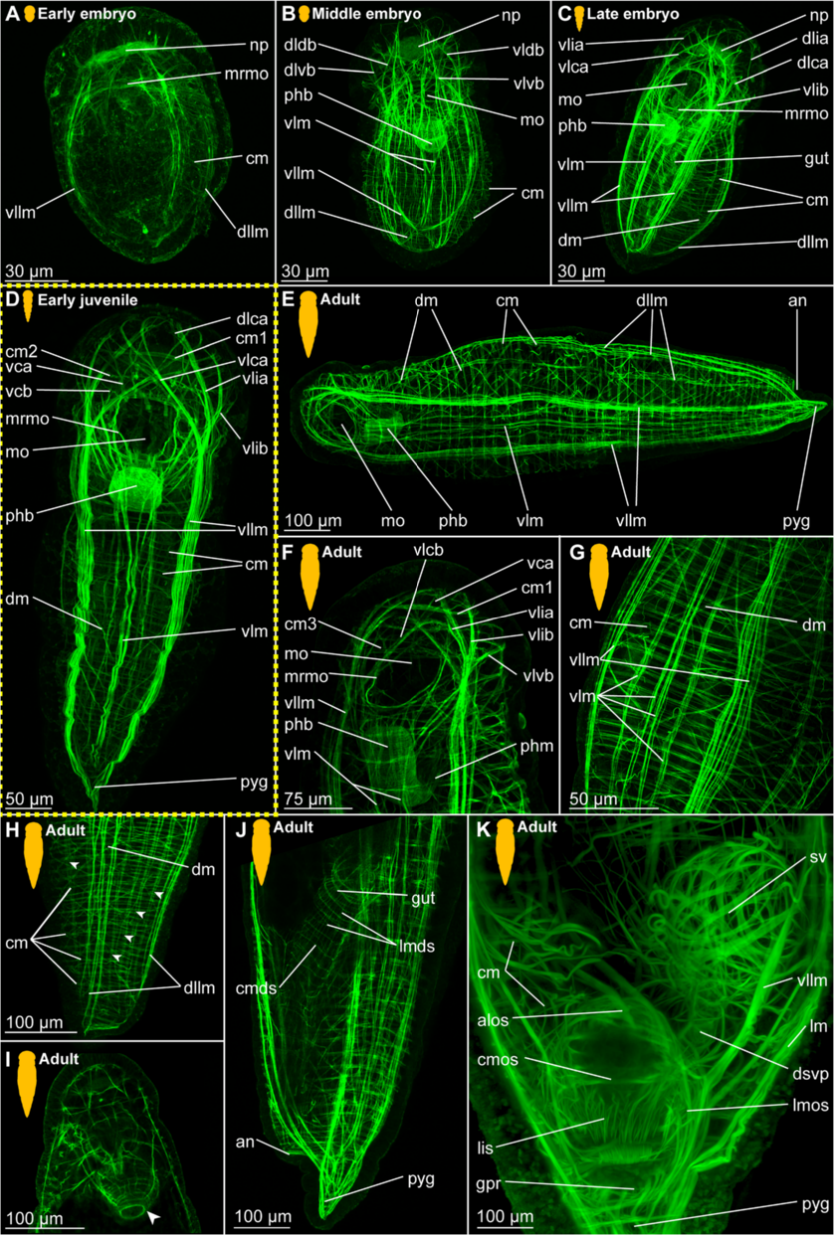
Myogenesis in *Dinophilus taeniatus*. Phalloidin-labelled actin-filaments shown in green, animals are oriented with the anterior end up (**a**-**d**, **f**-**k**) or to the left (**e**). Stages are indicated by silhouettes next to the figure capture, and the assignment to the respective stage next to them. The first signs of difference between the two species *D. gyrociliatus* and *D. taeniatus* are emphasized by a yellow dashed-lined frame around the picture. **a**-**c** embryonic development: **a** ventral view of an early embryo (5–6 days after eggs are deposited), **b** ventral view of a middle embryo (7–9 days after eggs are deposited), **c** ventral view of a prehatching embryo (10–14 days after eggs are deposited), **d** ventral view of an early juvenile animal, **e**-**k** adult worm: **e** ventrolateral view of the overall bodywall musculature, **f** detail of the head musculature in ventral view, **g** ventrolateral view of the body wall musculature in the trunk, **h** dorsolateral view of a virtually cropped stack of the caudal region of the body (arrowheads point at thin bundles of longitudinal muscles), **i** ventral view of the head (arrowhead points at basket-shaped musculature of the pharynx), **j** lateral view of the musculature in the digestive system in the posterior part of the body, **k** dorsal view of the copulatory organ and seminal receptacle. Abbreviations: alps – anterior loop of the penile sheath, an – anus, cm – circular muscle, cmds – circular muscle of the digestive system, cmps – circular muscles of the penile sheath, dlca – contralatero-anterior branch of the dorsolateral longitudinal muscle, dldb – dorsal branch of the dorsolateral longitudinal muscle, dlia – ipsilatero-anterior branch of the dorsolateral longitudinal muscle, dllm – dorsolateral longitudinal muscle, dlvb – ventral branch of the dorsolateral longitudinal muscle, dm – diagonal muscle, dsvp – muscular duct leading from the seminal receptacles to the penis, fmt – foregut-midgut transition, gpr – gonopore opening, lips – longitudinal inner muscles of the penile sheath, lm – longitudinal muscle, lmds – longitudinal muscle of the digestive system, lops – longitudinal muscles of the penile sheath, mo – mouth opening, mrmo – muscular ring around the mouth opening, np – neuropil, phb – pharyngeal bulb, phm – pharyngeal muscle, pyg – pygidium, sv – seminal vesicle, vca –contralatero-anterior branch of the ventral longitudinal muscle, vcb- – contralateral dorsal branch of the ventral longitudinal muscle, vlca – contralatero-anterior branch of the ventrolateral longitudinal muscle, vlcb – contralateral branch of the ventrolateral longitudinal muscle, vldb – dorsal branch of the ventrolateral longitudinal muscle, vlia – anterior ipsilaterial branch of the ventrolateral longitudinal muscle, vlib – ipsilatero-anterior branch of the ventrolateral longitudinal muscle, vllm – ventrolateral longitudinal muscle, vlm – ventral longitudinal muscle, vlvb – ventral branch of the ventrolateral longitudinal muscle

*Body wall musculature.* In contrast to *D. taeniatus*, where the multiple longitudinal muscle fibres can be seen spread along the entire body circumference (Fig. 5e, g, h), only six bundles are present in adult *D. gyrociliatus* females (one pair of dorsolateral, ventrolateral and ventral longitudinal muscles, Fig. 2h). All of them converge towards the posterior end of the body, where they seem to end blindly (Fig. 2h). Young adults can show a high number of diagonal muscles (dm, Fig. 2h), though this does not seem to be a fixed morphology.

*Prostomial musculature.* The muscles and their furcations as described in the juvenile stage get more defined (Fig. 2g).

*Musculature of the digestive system.* The gut musculature forms a thin layer of longitudinal and circular muscles (lmds, cmds, respectively, Fig. 2h). The latter are set further apart than the circular muscles of the body wall. The sigmoid muscle as described in females at hatching or juvenile stage extends ventrally in the hindgut, ending ventral close to the anus (sm, Fig. 2h).

The pharyngeal bulb is strongly connected to various muscles in the prostomial region, which are anchored in the epidermis of the prostomium (Fig. 2g). Additionally, the pharynx and the foregut are characterized by a series of circular muscle fibres positioned closely together, which cannot be observed in the posterior region of the digestive system.

#### *Dinophilus gyrociliatus* dwarf males

The onset of muscular development seems to be similar to the onset observed in females with longitudinal fibres emerging as two ventrolateral (vllm) pairs from the ventroanterior point of muscular origin (vpmo, Fig. 4a-c). In contrast to females, dwarf males do not develop a digestive system and a stomodeum could not be observed. We therefore used the formation of the anterior ciliary field (see below for a more detailed description) on the ventroanterior side of the animal as well as the pair of dorsomedian nephridia and the formation of the distinctive penile musculature as landmarks. During subsequent muscular growth and differentiation, the ventroanterior point of origin of muscles gets more refined and changes from an undefined mass (Fig. 4a, b) into the triangular form that can be observed in the adults (Fig. 4d, e). While the ventrolateral muscles line the lateral sides of the body, the dorsal fibres (dlm) extend dorsally as one bundle and bifurcate posterior to that (Fig. 4b, c). As they continue extending towards the posterior end of the body, where the copulatory organ is formed in subsequent steps, circular muscles (cm) are added in an anterior-to-posterior pattern (Fig. 4b, c). These structures consist of individual fibres, which emerge from the ventral side of the animal distal to the longitudinal muscle and extend further dorsally, until they fuse and form a ring (Fig. 4c-e), similar to the pattern seen in females (Figs. 2, 5).

The penile region is the most prominent and complex muscular part in the dwarf males. It comprises a penile sheath (psm) with an organized inner and a meshwork-like outer layer (Fig. 4d-i). These components start to form after the ventrolateral longitudinal muscles have extended to the posterior end of the body and at least four circular muscles are formed. The penile sheath (psm) is joined by ventrolateral longitudinal muscles, which develop loop-like structures prior to additional details of the penile musculature (Fig. 4d). The dorsal longitudinal muscles join the structure prior to the development of the penile cone (pc), which forms independently of the penile sheath (Fig. 4f, g). Subsequently, the fibres forming the sheath start to get more defined and link the musculature of the copulatory organ to the ventrolateral and dorsal muscles of the body wall (Fig. 4d). At the same time, all six circular fibres have formed and the male starts moving (i.e. stretching and compressing) inside the egg layer, using motile cilia and muscular contractions (approximately half a day to a day before hatching, Fig. 4d).

*Musculature of the copulatory organs in adults.* The innermost layer of the penile sheath is dominated by ten to twelve fibres, which are arranged in a horizontal pattern extending from the anteriormost onset of the penile sheath to the most posterior point (lips, Fig. 4f, h). These muscles are weakly labelled with phalloidin, especially when compared to the strong labelling of the penile cone musculature and the outer layer and tightly enclose the penile cone (Fig. 4f, g). In the posterior part, muscle fibres form a ring, which is adjacent to the gonopore (gpr, Fig. 4g).

The outer layer of the sheath consists of circular (cmps) and longitudinal muscle fibres (lops), which are separate from the muscles of the body wall (Fig. 4e-h). In the most anterior part, several projections radiate into the body, but their ends could not be traced successfully in all specimens. In contrast to the organized pattern observed in the inner sheath, muscles in the outer sheath have a network-like appearance. Most obvious is a posterior ring formed by the fusion of ventrolateral longitudinal muscles with the penile sheath (ros, Fig. 4i). Furthermore, this ring encloses another ring-like structure, which is formed by the inner sheath.

Each of the ventrolateral longitudinal muscles of the body wall forms a bifurcation anterior to the penile sheath, so two smaller bundles can fuse with the sheath on each side. While the thinner part of this bifurcation seems to fuse with the posterior ring, the more prominent part terminates lateral at the penile sheath after forming a loop on each side of the animal (vlps, Fig. 4f). The dorsal longitudinal muscles contribute to the outer sheath by forming loops laterodorsal at the external surface. These loops join at the approximate body midline (dlps, Fig. 4i). Furthermore, the longitudinal muscular strands extend and form an additional loop anterior to the anterior loop of the outer sheath before bifurcating and merging with the sheath (alps, Fig. 4f). The pattern observed on the dorsal side of the sheath is more complex than the one found ventrally. Individual muscle fibres emerging from the posterior muscle ring and extending towards the anterior of the body form the main part of the network. They are thereby connecting to the loops, similar to fibres emerging from the ventral towards the anteriodorsal side of the sheath.

#### Dinophilus taeniatus (both sexes)

##### Embryonic development

The first muscle fibres differentiate soon after gastrulation during early embryogenesis. Similar to female *D. gyrociliatus*, the neuronal f-actin is labelled in the developing neuropil, which is why phalloidin-labelled materal can be detected in the prostomium (Fig. 5a-c). In the prostomial region, the first muscular element to develop is a muscular ring around the mouth opening (mrmo, Fig. 5a). The main structures of the body wall are established short thereafter in early *D. taeniatus* embryos: paired ventrolateral (vllm) and dorsolateral longitudinal muscles (dllm) can be distinguished, as well as fragments of future circular muscles (cm) on the lateral sides of the embryo (Fig. 5a).

The muscular system develops very quickly: a large number of muscles characteristic for adults is already present in late embryos. The body wall consists of multiple longitudinal muscles, the majority of which are grouped into ventral, ventrolateral, and dorsolateral longitudinal muscles (Fig. 5b). However, there are also thin separate longitudinal muscle fibres. Numerous circular muscles develop, forming complete rings tracing the body circumference. They occupy the entire length of the body. Longitudinal muscles bifurcate in the prostomial region organizing the prostomial musculature: both ventrolateral and dorsolateral muscles produce dorsal and ventral branches in the same manner (vldb, vlvb, dlvb, dldb, Fig. 5b). The pharyngeal bulb found posterior to the mouth opening is the first element of the musculature of the digestive system (phb, Fig. 5b).

Prehatching embryos demonstrate a regular organization of the body wall musculature and a complex prostomial musculature. Separate longitudinal muscles, seen earlier in the body, appear to be closer to the main muscle strands. Nevertheless, the main longitudinal muscles obviously consist of a group of up to ten muscle fibres (Fig. 5c). Diagonal muscles join the grid of circular and longitudinal muscles (dm, Fig. 5c). More muscle branches originating from the ventrolateral and dorsolateral longitudinal muscles are registered in the prostomial region. As the digestive system differentiates, a defined muscle layer emerges in the gut wall (Fig. 5c).

##### Hatching & early juvenile stages

Additional muscle fibres are detected in the prostomium and trunk of *D. taeniatus* juvenile worms. Main thick longitudinal muscles of the body wall are accompanied by several thin longitudinal muscles. The prostomial musculature adds branches (namely contralatero-anterior and contralateral dorsal branches) of a ventral longitudinal muscle (vca, vcb, Fig. 5d). In addition to the body circular muscles, several circular muscles differentiate in the prostomial region as well (cm1, cm2, Fig. 5d).

##### Adult

*Body wall musculature.* In adults, the number of longitudinal muscles increases dramatically compared with the previous stages. Additional multiple bundles branch out from ventral and dorsolateral longitudinal muscles (Fig. 5e, g, h). Ventrolateral muscles appear to be the most condensed among the other longitudinal muscles (vllm, Fig. 5e, g). Thus, longitudinal muscular bundles number up to 14 in an adult *D. taeniatus*. This is different in *D. gyrociliatus,* where the number of longitudinal muscles in the body wall is not altered during maturation and which therefore presents six longitudinal muscular bundles in juveniles and adults (Fig. 2f, h).

*Prostomial musculature.* The prostomial musculature of adult *D. taeniatus* is quite similar to that of a juvenile; however, several branches show more bifurcations than earlier (Fig. 5f).

*Musculature of the digestive system.* The pharyngeal bulb is very well seen posterior to the mouth opening (phb, Fig. 5e, f). Pharyngeal muscles connect the bulb with the other muscles in this region of the body (phm, Fig. 5f). The basket-like muscle sack surrounds the pharynx (Fig. 5i). The gut wall demonstrates a net of numerous longitudinal and circular muscles (lmds, cmds, Fig. 5j).

No sigmoid muscle as observed in females of *D. gyrociliatus* is detected around the gut of *D. taeniatus* at any developmental stage.

*Musculature of the copulatory organs.* The musculature of the male reproductive system in *D. taeniatus* males supports one pair of seminal receptacles and the penis (with the penile cone and the penile sheath, Fig. 5k). Regardless of the size difference between specimens of the two species, we found the arrangement of the inner longitudinal (lops) and outer circular muscles (cpms) in the penile sheath show close resemblance (compare Figs. 4d-i, 5k). Additionally, the connection between the copulatory organ and the longitudinal body wall musculature are similar in these two species with regards position and arrangement of muscle fibres.

### Ciliary structures

#### Outer ciliary patterns (ciliary bands, ciliary fields)

##### Dinophilus gyrociliatus females

**Fig. 6:**
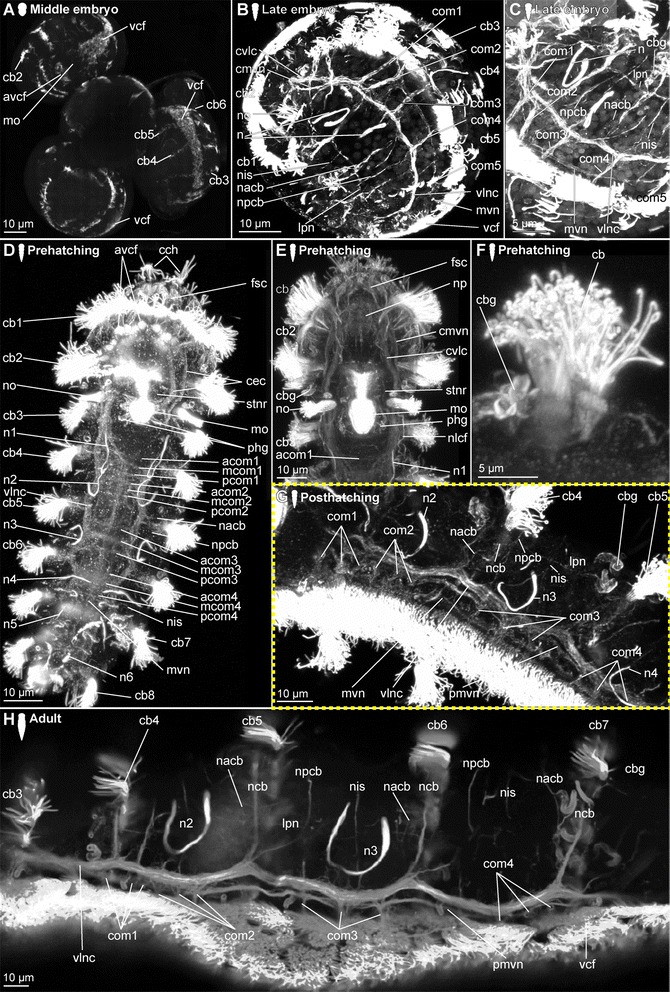
Development of ciliation patterns, nervous system and nephridia in *Dinophilus gyrociliatus* (depicted with acetylated α-tubulin-LIR). Acetylated α-tubulin-like immunoreactive filaments shown in white, animals are oriented with the anterior end up (**b**, **c**-**e**) or to the left (**f**-**h**). Several embryos within one cocoon are shown in **a**), with the anterior end down to the left (uppermost embryo) or down (lowest embryo to the right). Stages are indicated by silhouettes next to the figure capture, and the assignment to the respective stage next to them. The first signs of difference between the two species *D. gyrociliatus* and *D. taeniatus* are emphasized by a yellow dashed-lined frame around the picture. **a** Female embryos inside the cocoon (3.5–4 days after the eggs were deposited), **b** late embryos inside the egg layer and cocoon curled with the ventral side outwards showing the ventral nervous system, **c** detail of **b**) and the ventral and peripheral nervous system, **d** ventral view of a prehatching female with the nervous system, nephridia and ciliation pattern, **e** detail of the head and anterior part of the body, **f** detail of the ciliary band with cilary band glands, **g** lateral view of and early juvenile females with nephridia and nervous structures, **h** lateral view of the nervous and nephridial system in an adult female. Abbreviations: acom – anterior commissure, avcf – anterioventral ciliary field, cb1-8 – ciliary band 1–8, cbg – ciliary band gland, cch – compound cilia of the head, cmvn – circumesophageal commissure forming the medioventral nerve, com1-5 – commissure 1–5, cvlc – circumesophageal commissure forming the ventrolateral nerve cord, fsc – flask shaped cell, lcf – lateral ciliary field, lpn – longitudinal peripheral nerve, mcom – median commissure, mo – mouth opening, mvn – medioventral nerve, *n* = n1-6 – nephridium 1–6, nacb – lateral nerve anterior to the ciliary band, ncb – nerve of the ciliary band, nis – intersegmental lateral nerve, nlfc – nerve innervating the lateral ciliary field, npcb – lateral nerve posterior to the ciliary band, pcom – posterior commissure, phg – pharyngeal gland, pmvn – paramedioventral nerve, vcf – ventral ciliary field, vlnc – ventrolateral nerve cord

*Embryonic development*. The onset of the development of the ciliary patterns is approximately 24–36 h after the cocoons have been deposited, when the first (incomplete) transverse ciliary band forms at approximately one third of the body length. This ciliary structure consists of symmetrical lateral bands on both sides of the ventral mouth opening (mo), and subsequently extend dorsally (Fig. 6a, b). After the formation of the second prostomial ciliary band (as also described by [55]), the anteriormost (cb1) as well as all posterior ciliary bands (cb3-cb8) develop almost simultaneously. Each ciliary band starts as lateral formation, and later on extends dorsally (Fig. 6a-c). The ventral ciliary field (vcf, Figs. 6a, b) precedes the development of the ciliary bands 1 and 3–7 and extends further posterior during subsequent development. The ciliary structures were observed to contribute to the rotational movements these animals show before hatching.

**Fig. 7:**
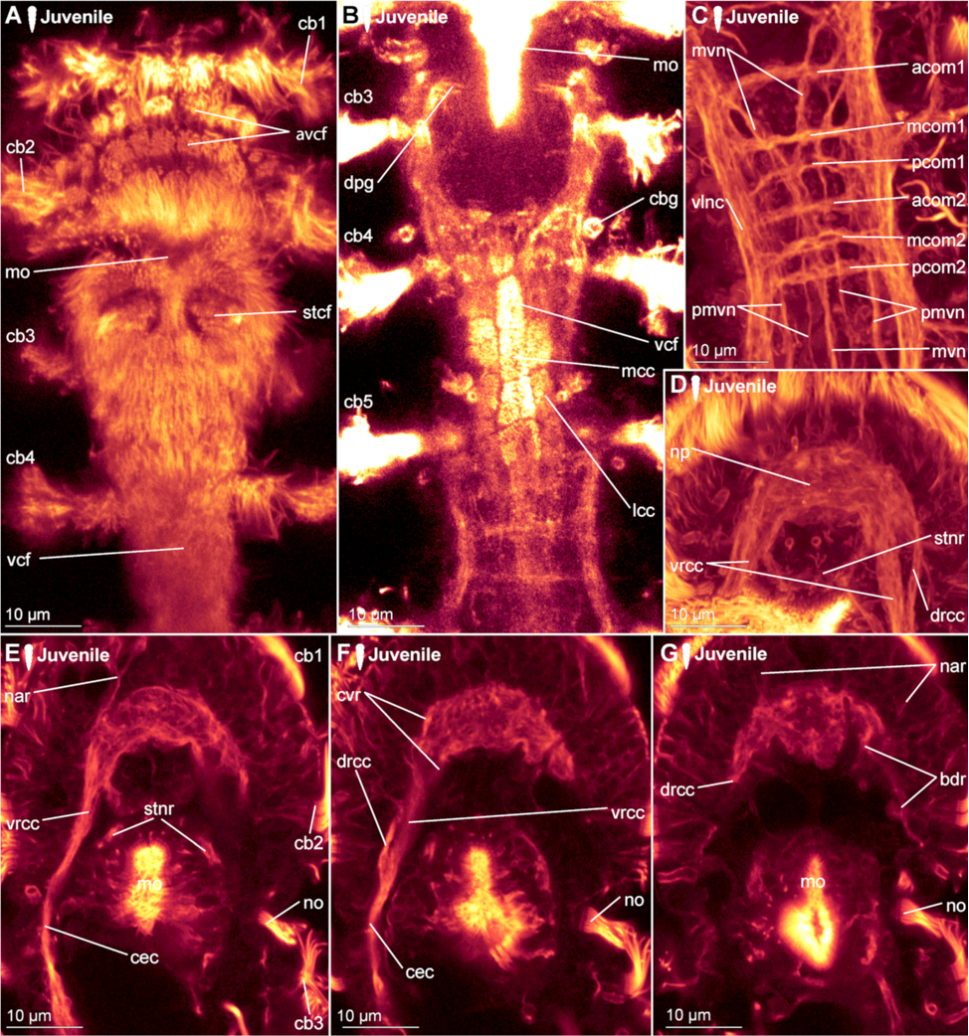
Correlation between outer ciliary and nervous structures in early juvenile females of *Dinophilus gyrociliatus* and details of the neuropil. Acetylated α-tubulin-like immunoreactive filaments shown in”glow”, animals are oriented with the anterior end up. **a** Ventral ciliary field of a juvenile female with spot-like multiciliated cells (svcf), stomatogastric ciliary fields (stcf) and more elongated multicilited cells (lcc), **b** multiciliated cells along the ventral body side, **c** detail of the anterior region of the ventral nervous system with the commissural sets, **d** detail of the neuopil with the dorsal and ventral root of the circumesophageal connective, **e**-**g** sections through the neuropil from the ventral to the dorsal side, showing the commissures of the ventral and dorsal root: **e** section through the ventral root with the base of the circumesophageal connective, **f** condensed fibres within the ventral root of the circumesophageal connective, **g** section through the dorsal root. Abbreviations: acom – anterior commissure, avcf – anteroventral ciliary field, bdr – branches of the dorsal root, cb – ciliary band, cbg – ciliary band gland, cec – circumesophageal connective, cvr – commissures of the ventral root of the circumesophageal connective, dpg – ducts of the pharyngeal glands, drcc – dorsal root of the circumesophageal connective, lcc – lateral multiciliated cell of the ventral ciliary field, mcc – median multiciliated cell of the ventral ciliary field, mcom – median commissure, mo – mouth opening, mvn – medioventral nerve, nar – nerves innervating the anterior rim, pcom – posterior commissure, pmvn – paramedioventral ventral nerve, stcf – stomatogastric ciliary field, stnr – stomatogastric nerve ring, svcf – stomatogastric ventral ciliary field, vcf – ventral ciliary field, vlnc – ventrolateral nerve cord, vrcc – ventral root of the circumesophageal connective

*Hatching & early juvenile stages.* In hatching animals, the ciliary bands of the body were observed to be nearly complete, except for the space taken up by the ventral ciliary field. Each ciliary band is traced by a series of tubular glands (cbg, Fig. 6f). The two transverse ciliary bands of the prostomium (cb1 and cb2) remain incomplete on the dorsal side similar to adults (data not shown). They also join the dense ventral ciliary field develops anterior to the mouth opening (avcf, Figs. 6d, 7a). The ventral ciliary field in this region is constituted by individual small cells, which are arranged in a semicircle in two rows of eight (the row closer to the mouth opening) to ten (the anterior row) cells (indicated by arrowheads in Fig. 6d). Six to eight relatively big, elongated, flask-shaped cells (fsc, Figs. 6d, e, 7f, g) can be found adjacent to the compound cilia (cch, Figs. 6D, e, 7e-g) on the dorsoanterior side of the prostomium. Their ducts extend ventroposterior and end posterior to the neuropil (dfsc, Fig. 7g). They can also be detected in late embryos and hatchlings. Next to the sensory compound cilia in the anterior part (cch) and the eyes (e) on the dorsal side of the prostomium, a pair of narrow ciliary fields is located lateral on the first segment (lcf, Figs. 6d, e).

The main portion of the ventral ciliary field is constituted by six elongated, multiciliated cells per row. They seem to fuse in the middle of the trunk to form one median line embraced by one additional row on each side (Fig. 7b). It extends from the mouth opening to the posterior end of the body, being broad in the region of the mouth opening and getting narrower towards the posterior (Fig. 7b). Posterior to the T-shaped mouth opening, paired regions of the ventral ciliary field probably serves as additional sensory organ (stcf, Fig. 7a) and demonstrates a different arrangement (orientation of cilia, shape of the cell, Fig. 7a).

*Adult.* Adult animals are characterized by a dense, complete ciliation on the ventral side of the animal (vcf, Fig. 6h), extending from anterior of the mouth opening towards the posterior tip of the animal, including the pygidium. The number of ciliary bands does not increase and therefore equals seven in adult females like in juveniles (data not shown). The ciliary bands probably support swimming behaviour over short distances in these animals.

##### Dinophilus gyrociliatus dwarf males

**Fig. 8:**
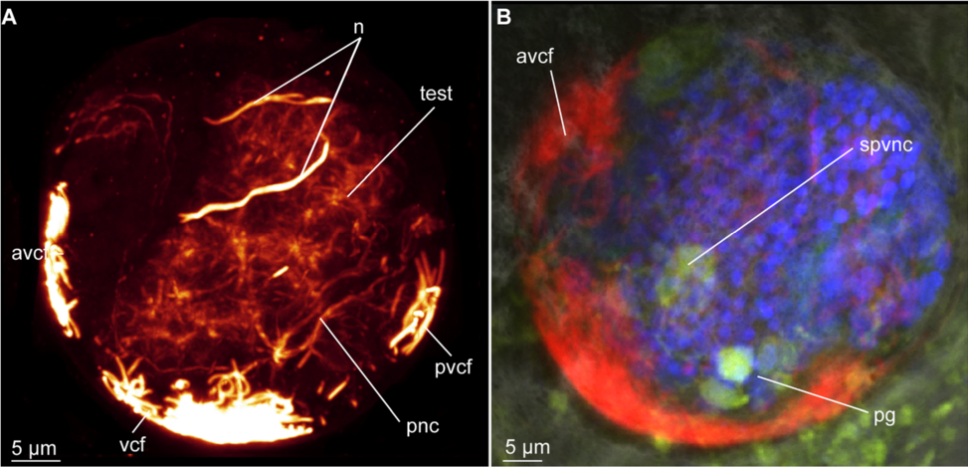
Nervous system in dwarf males of *Dinophilus gyrociliatus*. Animals are oriented with the anterior end to the left, **a** Acetylated α-tubulin-like immunoreactive filaments shown in “glow”, indicating one pair of protonephrida and the individual ciliary fields, **b** acetylated α-tubulinergic-like immunoreactive filaments shown in red, serotonin-like immunoreactive perikarya and fibres in yellow, labelling of DNA with DAPI in blue with focus on the penile ganglion. Abbreviations: avcf – anterioventral ciliary field, n – nephridium, pg – penis ganglion, pnc – penis nerve cord, pvcf – posterior ventral ciliary field, spvnc – serotonin-like immunoreactive perikarya of the ventral nerve cord, test – testis, vcf – ventral ciliary field

Dwarf males have three ciliary fields, which can be found in the anterior (avcf), the posterior (pvcf) and the ventral side (vcf) of the animal, sometimes giving the appearance of a continuous band, though constituted by separate cells (see also [56, 57], Fig. 8a, b).

##### Dinophilus taeniatus (both sexes)

**Fig. 9:**
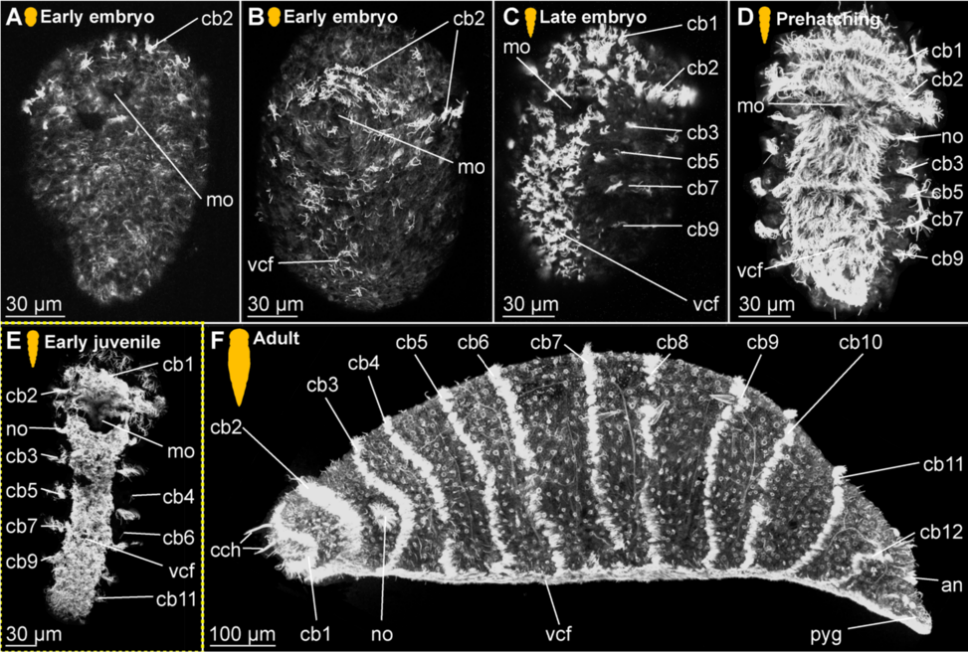
Development of external ciliation patterns in *Dinophilus taeniatus*. Acetylated α-tubulin-like immunoreactive filaments shown in white, animals are oriented with the anterior end up (**a**-**e**) or to the left (**f**). Stages are indicated by silhouettes next to the figure capture, and the assignment to the respective stage next to them. The first signs of difference between the two species *D. gyrociliatus* and *D. taeniatus* are emphasized by a yellow dashed-lined frame around the picture. **a**-**d** embryonic development: **a** ventral view of an early embryo (5–6 days after eggs have been deposited), **b** ventral view of embryo at ventral ciliary field stage, **c** ventral view of a late embryo with the onsets of the developing ciliary bands 1, 3, 5, 7, and 9, **d** ventral view of a prehatching embryo with well developed external ciliation, **e** ventral view of an early juvenile specimen before duplication of the ciliary bands, **f** lateral view of an adult specimen, with well-developed duplication of ciliary bands. Abbreviations: an – anus, cb1-12 – ciliary band 1–12, cch – compound cilia of the head, lcf – lateral ciliary field, mo – mouth opening, pyg – pygidium, vcf – ventral ciliary field

*Embryonic development.* The first external ciliary structure to develop is a transverse ciliary band lateroanteriorly to the stomodeum (cb2, Fig. 9a). This ciliary band can be detected as early as 5–6 days after oviposition and corroborates previous findings by Nelson [55], who suggested this ciliary band to be possibly homologous to the prototroch in planktonic trochophore larvae of other annelids. A few days later, the ciliary field forms at the ventral surface posterior to the stomodeum (vcf, Fig. 9b) and fuses lateral to the stomodeum with the previously formed transverse ciliary band. On day 8–9 of embryonic development, additional transverse ciliary bands start to develop parallel and anterior (cb1) as well as posterior (cb3, 5, 7, 9) to the first one (cb2, Fig. 9c). While they represent only ciliary stripes on the ventrolateral sides of the embryo at the onset of ciliary development, the dorsal gaps are closed during succeeding developmental steps. The anteriormost ciliary band (cb1) develops in the middle region anterior to the stomodeum and then extends laterally towards the dorsal surface. Later on during development all these ciliary structures become more prominent. The lateral ciliary fields (lcf) emerge ventral to the mouth opening at both sides of the prehatching embryo (Fig. 9d). The ventral ciliary field is wide enough to cover almost the entire ventral surface when four ciliary bands posterior to the stomodeum are continuous structures on the dorsal sides.

*Hatching & early juvenile stages.* The juveniles are larger than the embryos as they start feeding and elongate. They still possess the wide ventral ciliary field and seven transverse ciliary bands embracing their bodies (Fig. 9e), while the additional ciliary band per segment starts to develop due to duplication, which is also mirrored by the nervous system (see below).

*Adult.* Adult animals are characterized by the most developed ciliary coverage, which is used for swimming and gliding locomotion. Two pairs of compound cilia are well represented at the anterior end of the animal (cch, Fig. 9f). The two anteriormost ciliary bands are visible anterior the stomodeum and remain incomplete on the dorsal side. Unlike *D. gyrociliatus*, the number of ciliary bands located posterior to the stomodeum doubles during maturation of *D. taeniatus*, so an adult worm has a total of 12–14 ciliary bands. The ventral ciliary field extends from the mouth opening till the posterior tip of the worm.

#### Protonephridia

##### Dinophilus gyrociliatus females

*Embryonic development.* The first nephridial pair can be detected belatedly after formation of the trunk ciliary bands. Either due to spatial or developmental constrains, the first pair of protonephridia is not elongated, but bent, with the terminal cell being close to the body wall of the animal as well as close to the opening to the outside. Before hatching, five of the six pairs of nephridia develop, with the more posterior ones being more elongated and straight (n, Figs. 6b, c).

*Hatching & early juvenile stages.* After hatching, a sixth pair of nephridia is added posteriorly in the animal, close to the pygidium (Fig. 6d, g). With further growth, the ducts of especially the anteriormost nephridial pairs extend towards the lateral sides. The posterior pairs, however, remain strictly constricted to the ventral side of the animals.

*Adult.* The nephridia have an average length of 45 μm (the posteriormost pair is the longest). The first three pairs are u-shaped, with the terminal cells located closely to the nephridiopores. The anteriormost pair is located closer to the fourth ciliary band and therefore nearly centred in the second body segment, the successive two pairs are found situated between the ciliary bands of segment 3 and 4, and closer to the posterior end of the segmental borders (Fig. 6h). This is similar to the pattern found in the forth and the fifth nephridial pair. However, these are found on the ventral side of the body, while the first three are located lateroventrally. The fourth pair is more curved and bent, and can also be found closer to the body midline on the ventral side. The fifth pair is relatively straight, with the terminal cell being close to the ventrolateral side of the animal and the nephridiopore close to the body midline (Fig. 6h). It is the longest nephridium, spanning approximately 55 μm in length. The sixth pair of protonephridia is shorter (30–35 μm) and hard to detect due to being surrounded by the dense ciliary brush of the ventral ciliary band.

##### Dinophilus gyrociliatus dwarf male

One pair of protonephridia (n, Fig. 8a) is found in the dwarf male. In contrast to the female nephridia, which are exclusively ventral or ventrolateral (Fig. 6), this nephridial pair is dorsal and located in the anterior third of the body (Fig. 8a).

##### Dinophilus taeniatus (both sexes)

**Fig. 10:**
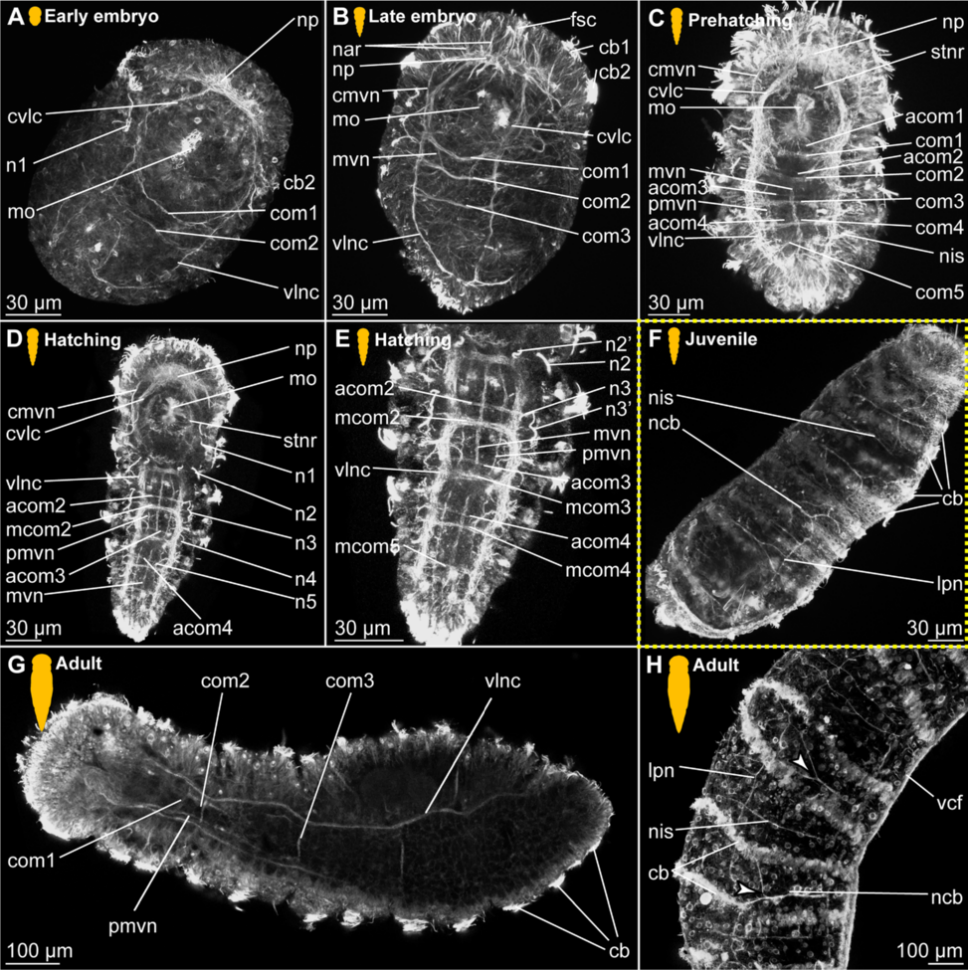
Development of the nervous system in *Dinophilus taeniatus*. Acetylated α-tubulin-like immunoreactive filaments shown in white, animals are oriented with the anterior end up (**a**-**f**, **h**) or to the left (**g**). Stages are indicated by silhouettes next to the figure capture, and the assignment to the respective stage next to them. The first signs of difference between the two species *D. gyrociliatus* and *D. taeniatus* are emphasized by a yellow dashed-lined frame around the picture. **a**-**c** embryonic development: **a** ventral view of an early embryo, note a dumbbell like shape of the neuropil, **b** ventral view of a late embryo, **c** ventral view of a prehatching embryo, **d** ventral view of the central nervous system in an early juvenile, **e** detail of the posterior part of the nervous system in an early juvenile, note the peculiar sets of acetylated α-tubulin-like immunoreactive structures forming the protonephridia, **f** left lateral view of a juvenile specimen with details of the peripheral nerves, **g**, **h** adult: **g** ventral view of the major parts of the ventral nervous system, **h** lateral view of the trunk of an adult specimen with details of peripheral nerves, note bifurcations (indicated by arrowheads) of ciliary band nerves. Abbreviations: acom – anterior commissure, cb – ciliary band, cmvn – circumesophageal commissure forming the medioventral nerve, com1-5 – commissure 1–5, cvlc – circumesophageal commissure forming the ventrolateral nerve cord, fsc – flask-shaped cells, lpn – longitudinal peripheral nerve, mcom – median commissure, mo – mouth opening, mvn – medioventral nerve, n1-5 – nephridium 1–5, n2′, n3′ additional part of respective nephridium, nar – nerves innervating the anterior rim, ncb – nerve of the ciliary band, nis – intersegmental lateral nerve, np – neuropil, pmvn – paramedioventral nerve, stnr – stomatogastric nerve ring, vcf – ventral ciliary field, vlnc – ventrolateral nerve cord

*Embryonic development.* Protonephridia are formed in anterior-posterior direction during embryogenesis. The anteriormost pair of protonephridia develops simultaneously with the first transverse ciliary band lateral to the stomodeum (n1, Fig. 10a). Additional protonephridia are formed in loose correlation to the transverse ciliary bands (Fig. 10b, c). Therefore, protonephridia cannot be used as reliable segmental markers as in most other polychaetes [80–82].

*Hatching and juvenile stages.* Prehatching embryos have four pairs of protonephridia, and juveniles have five pairs of protonephridia (Fig. 10d) (in contrast to five nephridial pairs in late embryos of *D. gyrociliatus*, Figs. 6d, g). However, their position does not correspond to other regular structures (such as ciliary bands, transverse neural commissures, etc.). It should also be noted, that *D. taeniatus* protonephridia differ in shape (or number?) from those of *D. gyrociliatus*: instead of u-shaped protonephridia of *D. gyrociliatus* (Fig. 6d), *D. taeniatus* has two separate branches each about 15–25 μm long (n2′, n2, n3′, n3, Fig. 10e). It is not clear whether there are two protonephridia on each side (in this case, a prehatching embryo has four pairs of combined protonephridial pairs) or the median part of one protonephridium is not immunoreactive with antibodies directed against tubulin structures (or does not have tubulin structures?). However, the pattern in *D. taeniatus* resembles observations in *Trilobodrilus sp.*, another member of Dinophilidae [11]. In any case, this is a significant difference between the two morphotypes investigated here.

*Adult.* The number of nephridia does not change during maturation, whereby adult worms are characterized by five pairs of protonephridia (data not shown) in contrast to six pairs in adult *D. gyrociliatus* females (Fig. 6h). However, at this stage acetylated α-tubulin-LIR is difficult to detect in the delicate internal structures because of the strong immunoreactivity of the external ciliation.

### Nervous system shown with acetylated α-tubulin-LIR

#### *Dinophilus gyrociliatus* females

##### Embryonic development

Since the early development of the nervous system is covered in high detail in a forthcoming study (Fofanova et al., in prep), the development patterns described here focus on the late embryonic stages (Figs. 6b-d).

In the late embryo, the neuropil (np) is well developed and located central in the prostomium, with individual nerve fibres extending towards the anterior rim of the body, probably innervating the anterior rim of the prostomium (data not shown). As described in [11], a dorsal and a ventral root of the circumesophageal connective can be distinguished, which lead posterior before seemingly fusing at approximately the level of the nuchal organs (lateral ciliary fields, lcf, Fig. 6b). There, they form the uniform circumesophageal connectives, which embrace the mouth opening and pharyngeal area, before forming the pair of prominent ventrolateral longitudinal (vlnc) and the pair of medioventral (mvn) nerve cords (Figs. 6b, c).

The ventral nervous system consists of two ventrolateral nerve cords (vlnc), and one pair of incompletely fused median nerve cords (mvn, Fig. 6b, c), all of which extend to the posterior end of the animal. A distinct pattern of one thick commissure per segment is detected in the ventral nervous system in late embryogenesis (com1-5, Fig. 6b, c).

Several thin, segmental and lateral nerves were found in this study emerging from the ventral nervous system towards the dorsal side of the body (nerves tracing the ciliary band - ncb, nerves anterior to the ciliary bands - nacb, nerves posterior to the ciliary bands - npcb and intersegmental nerves - nis, Fig. 6b, c). These nerves partly fuse laterally in later life stages, but are well separated in late embryos. In contrast to Müller & Westheide [11], who found them to be limited to the central region of the trunk, this study found the pattern continuous from the third to the eighth ciliary band. Further peripheral longitudinal nerve fibres (lpn) extend perpendicular and external to the transverse nerve fibres towards the posterior end of the body. They can be seen lateral and dorsal in the body (Fig. 6b, c).

##### Hatching & early juvenile stages

*Central nervous system.* The brain is constituted by approximately 600–750 nerve cells (counted from DAPI-staining), with their perikarya forming a sheath surrounding the neuropil, which is more prominent on the anterior, lateral, posterior and dorsal sides, but rather thin (one incomplete layer) on the ventral side (data not shown). Although the neuropil appears uniform, a dorsal and a ventral root of the circumesophageal connective with several condensed nerve fibre bundles in both roots can be distinguished (Fig. 7d-g). Individual nerves emerge from both roots towards the anterior rim of the prostomium with the compound cilia (Figs. 6e, 7e, g), while no distinct tracts are linked to the dorsal, sunken-in eyes. Although the main portion of the ventrolateral nerve cords (the most prominent cords of the ventral nervous system) emerges from the anterior dense nerve bundle of the ventral root, at least three further ramifications are detected in the ventral root and additional strands also extend into the dorsal root (Fig. 7f, g). Both roots seemingly fuse posterior to the brain, at the approximate median level of the mouth opening (Fig. 7f). The median (mvn, Figs. 6d, 7c) and paramedioventral nerve cords (pmvn, Fig. 7c) are formed by thin nerve bundles branching off from the ventrolateral nerve cords towards the body’s midline. While the median pair of the paramedioventral nerves is more prominent, the thinner, more lateral pair regularly runs closer to the adjoining nerve bundles (Fig. 7c). The patterns of the ventral nerves and of the ciliated cells in the ventral ciliary field (described above) seem to be correlated, possibly indicating the close relationship between these two systems (Fig. 7b). Therefore we found seven longitudinal nerve bundles in the ventral nervous system, as was indicated by [11]. The two pairs of paramedioventral nerves of *D. gyrociliatus* in contrast to the findings in *Trilobodrilus* sp. [11] and *D. taeniatus* possibly resemble one split bundle of nerve fibres in the latter.

The lateral ciliary fields (nuchal organs) are innervated by thin nerves emerging from the circumesophageal commissure (nlcf, Fig. 6e). At the level of the mouth opening, a pair of thicker nerve bundles extends from the mouth epithelial cells or the surrounding pharyngeal glands laterally to the ventrolateral longitudinal nerve cords, though it could not be traced where they end (Fig. 7b). To what extend the nervous plexus, which can best be detected with serotonin-LIR ventral to the ventral nervous system, is connected to the neuropil or the ventral cords could not be ascertained in this study.

One additional nerve bundle is detected anterior and posterior to each prominent transverse commissure when the females hatch, resulting in a moderately thick anterior (acom), the most prominent median (mcom) and the thin posterior transverse nerve bundles (pcom, Figs. 6d, g, 7c) as described previously [11]. This overall pattern is best exemplified in the segments three and four, while there are some alternations in the most anterior and posterior segments (Fig. 6d, g). The triple-pattern of commissures of the anterior two segments is altered, since the commissural sets are drawn together and can therefore only be detected as sequence of six commissures, which are spaced equally apart (Fig. 6d). In the most posterior segment, only two commissures are formed (Fig. 6d). None of the commissures are arranged correlated with the ciliary bands or the associated nerves.

*Additional (dorsal, lateral, segmental) nerves.* The most prominent lateral nerves emerge from the ventrolateral nerve cord and trace the ciliary bands, consisting of four to five individual fibres (ncb, Fig. 6g). These nerves, which are 5–10 μm anterior to the ciliary bands, are parallel to a thinner bundle consisting of only one to two fibres, which are located posterior to the cells of the ciliary band (npcb, Fig. 6g). Another set of nerves is formed anterior to the ciliary bands (nacb, Fig. 6g, h). Their parallelism is retained throughout the entire circumference of the animal. Another nerve fibre extends further posterior and fuses with another nerve fibre in the intersegmental area of the animal (nis, Fig. 6d, g). Seven longitudinal nerves can be detected at periphery: one unpaired dorsal one, one pair of dorsolateral and two pairs of lateral nerves (lpn, Fig. 6g, h), extending throughout the body parallel to the cords of the ventral nervous system.

##### Adult

*Brain.* The compact neuropil is surrounded by a massive (approximately 10 μm) layer of nuclei on its anterior, dorsal, posterior and lateral side and a thinner layer on the ventral side (data not shown). A series of fibres emerges from the central neuropil and possibly innervates the compound cilia on the anteriormost tip of the animal as well as some other ciliated and non-ciliated cells (nar, Fig. 7e, g). Although the distinction into the roots of the circumesophageal connective and its commissures is less clear than in some of the hatching or juvenile animals, the dorsal and ventral root can be distinguished, however, the number of dense nerve fibre bundles connecting the root of either side of the body of especially the ventral root could not be ascertained in all investigated specimens. While the general layout of the ventral nervous system remains the same between hatchlings and adults, the neuropil seems to get denser. Like in juveniles, several fibres emerge dorsally and give rise to the unpaired dorsal and paired dorsolateral nerve fibres extending throughout the entire body (lpn, Fig. 6g, h).

The eyes are sunken deep inside the epidermis and come to rest inside the layer of nuclei around the neuropil (data not shown), so it is not clearly determined how many nerve fibres from which region connect to them.

*Ventral nervous system.* All eight nerve strands (one pair forming the median nerve by fusion, two pairs of paramedioventral nerves and a ventrolateral pair of main nerves) are connected by 18 commissures (com1-6, tcom), which are spread throughout the entire length in groups of three (segments one to four) and two commissures (fifth and sixth segment, data not shown for structures with alpha α-tubulin LIR, but it is shown below with neurotransmitter LIR). Although most likely associated with segments, they are located in the intersegmental region rather than within specific segments (Fig. 6h). As already demonstrated in the hatching and juvenile specimens, this pattern is further altered in the anteriormost segments, where six transverse fibre bundles are formed in close vicinity, possibly representing the commissures of the first and second segment. In the most posterior end, two commissures are positioned adjacent to each other: the dorsal subrectal and the ventral terminal commissure. Both of them are clearly located in the sixth segment, anterior to the pygidium and close to the last pair of protonephridia.

*Additional (dorsal, lateral, segmental) nerves.* Though carrying eggs can dilate the body wall and therefore obscure the pattern, the longitudinal and transverse peripheral nerves can be traced as continuous structures from the anterior into the posterior part of the body (Fig. 6h), as described in juvenile animals. In adult females, it can be clearly observed that the nerves are intraepidermal and therefore external to the body wall musculature.

*Stomatogastric nervous system.* The stomatogastric nervous system is constituted by a nerve ring around the esophagus originating from the dorsal root of the circumesophageal connectives (stnr, Fig. 7e).

#### *Dinophilus gyrociliatus* dwarf males

Acetylated α-tubulin LIR does not give sufficient signal for the description of individual nerve cells or cell connections in the dwarf male. Only the most prominent nervous connections, such as the penis nerve cord (pnc, Fig. 8a), connecting the ventral ganglion to the penis ganglion can be detected.

#### *Dinophilus taeniatus* (both sexes)

##### Embryonic development

Like in *D. gyrociliatus*, the main structures of the definitive central nervous system differentiate during embryogenesis in *D. taeniatus*. The neuropil (np) with the characteristic dumbbell shape and the ventral nervous system can be detected soon after gastrulation. The processes of the neuropil connect the already formed ventral root of the circumesophageal connective with a pair of longitudinal ventrolateral nerve cords (vlnc, Figs. 10a). Transverse commissures develop in rostro-caudal direction. In early neurogenesis, only the anteriormost one or two commissures can be detected. Later on, these structures are joined by additional neurite bundles, and also the neuropil changes its shape from dumbbell-shaped into oval. Several elongated flask-shaped cells (fsc) emerge in the ectoderm layer anterior to the neuropil. Thin separate neurites (nar) extend from the neuropil towards the anterior edge of the embryo. During further development, the number of commissures increases, while the anterior part of median nerves (mvn, Fig. 10b) differentiates between the first and second commissure.

Shortly before hatching, *D. taeniatus* embryos demonstrate a full set of longitudinal nerve cords of the ventral nervous system, consisting of one pair of ventrolateral, one pair of paramedioventral, and one unpaired median nerve cord (Fig. 10c). The latter originates from both ventrolateral nerve cords at the level of the second transverse commissure and converges towards the body midline at the level of the third commissure, from where it continues as one longitudinal cord towards the posterior end. It is assumed that its two components do not only arise by the ventrolateral nerve cords, but originate initially from the brain and form a loose bundle with the nerves of the ventrolateral nerve cords before separating again. Upon hatching, the ventral nervous system contains five commissures, however, an additional thin commissure is found anterior to the main thick commissures at this stage (Fig. 10c).

The stomatogastric nerve ring develops around the pharynx (stnr, Fig. 10c) and thin nerve fibres (intersegmental lateral nerves - nis), extend dorsally from the ventrolateral nerves between the transverse commissures.

##### Hatching & early juvenile stages

*Central nervous system.* The general pattern of the nervous system in prehatching embryos is similar to that of juvenile worms. The brain does not differ significantly from earlier stages; the ventral nervous system includes paired ventrolateral and paramedioventral nerve cords, the unpaired median cord, and five thick transverse commissural bundles (Figs. 10d). Each of the thick commissures is accompanied by an additional thin anterior neurite bundle, which develop at prehatching to hatching stage (acom, Fig. 10c-e).

*Additional (dorsal, lateral, segmental) nerves.* Intersegmental nerves extend laterally from the ventrolateral nerve cords between the ciliary bands (nis, Fig. 10f). They furcate and give rise to several thin branches, some of which appear to be joining the dorsolateral longitudinal nerves (lpn), whereas others extend further dorsally. All these nerves are very thin and consist of only few neural processes.

The stomatogastric nerve ring is more prominent than in previous stages and short commissures connect it to the ventral root of the neuropil (Fig. 10d).

##### Adult

Acetylated α-tubulin-LIR fails to reveal brain structures in adult *D. taeniatus*, since they hardly visualize nerve elements in the body of adult worms. Certain structures are nevertheless registered. Nerve bundles of the circumesophageal commissure organizing ventrolateral and paramedioventral nerve bundles are seen emerging from the head (vlnc, pmvn, Fig. 10g). Ventrolateral nerves and several commissures can be distinguished in the ventral nervous system. All revealed commissures are presented as only one transverse bundle, indicating that thin and main commissures probably condense during maturation.

As the number of ciliary bands doubles during maturation (Fig. 9f), lateral nerves underlying one ciliary band during earlier stages bifurcate in the lateral or dorsolateral region of the body, forming two branches (anterior and posterior) each of which innervates a respective ciliary band of a segment (ncb, Fig. 10h). Intersegmental nerves are found between segments, but they are thinner than the nerves of ciliary bands and do not furcate (nis, Fig. 10h). The lateral nerves cross a dorsolateral nerve (lpn) and extend further to the dorsal side as multiple very thin fibres.

### Nervous components showing neurotransmitter-immunoreactivity: serotonin-LIR

#### *Dinophilus gyrociliatus* females

##### Embryonic development

**Fig. 11:**
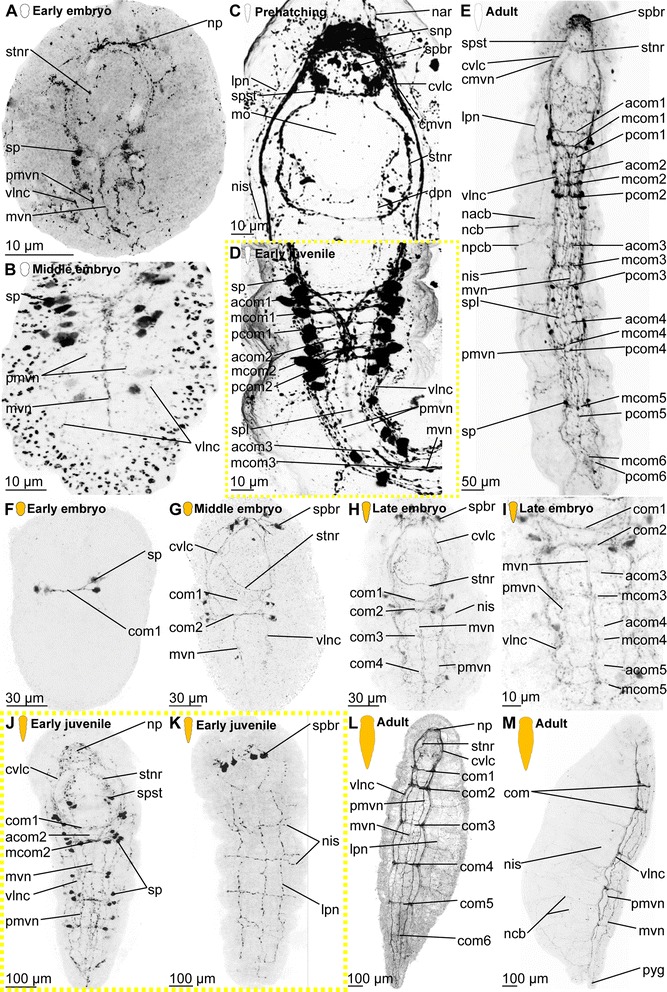
Neurogenesis in female *Dinophilus gyrociliatus* (**a**-**e**) and *D. taeniatus* (**f**-**m**) as revealed by serotonin-LIR. Serotonin-like immunoreactive elements shown as black structures on white background, animals are oriented with the anterior end up. Stages are indicated by silhouettes next to the figure capture, and the assignment to the respective stage next to them. The first signs of difference between the two species *D. gyrociliatus* and *D. taeniatus* are emphasized by a yellow dashed-lined frame around the picture. **a**, **b** embryonic development: **a** ventral view of the first indication of the serotonin-like immunoreactive nervous system in early embryos of *D. gyrociliatus* with perikarya forming along the ventral nervous system, **b** ventral view of a detail of the ventral nervous system with governing perikarya in ventral ciliary field stage, **c** ventral view of the head in a juvenile female, **d** ventral view of a detail of the nervous system in the anterior trunk segments in a hatching female, **e** ventral view of the serotonergic nervous system in an adult female, **f**-**j** embryonic development: **f** ventral view of an embryo at 1 ciliary band stage with first immunoreactive perikarya being located near the first commissure, **g** ventral view of an embryo at ventral ciliary field stage with four perikarya being detected in the head region, **h** ventral view of a late embryo, **i** detail of **h**) of the ventral nervous system, **j** ventral view of a juvenile specimen, **k** dorsal view of a juvenile with four perikarya dorsal to the neuropil, **l** ventral view of an adult specimen displaying a regular ladder-like organization of the ventral nervous system, **m** lateral view of an adult specimen. Abbreviations: acom – anterior commissure, cmvn – circumesophageal commissure forming the medioventral nerve, com1-5 – commissure 1–5, cvln – circumesophageal commissure forming the ventrolateral nerve cord, dpn – dorsal peripheral nerves, lpn – longitudinal peripheral nerve, mcom – median commissure, mo – mouth opening, mvn – medioventral nerve, nacb – lateral nerve anterior to the ciliary band, nar – nerves innervating the anterior rim, ncb – nerve of the ciliary band, nis – intersegmental lateral nerve, npcb – lateral nerve posterior to the ciliary band, pcom – posterior commissure, pmvn – paramedioventral nerve, snp – serotonin-LIR portion of the neuropil, sp – serotonin-LIR perikaryon, spbr – serotonin-LIR perikaryon of the brain, spl – serotonin-LIR plexus, spst – serotonin-LIR perikaryon of the stomatogastric nerve ring, stnr - stomatogastric nerve ring

The first serotonin-like immunoreactive neurons differentiate in the ventral region after several ciliary bands are formed; at approximately the same time the first sign of FMRFamide-LIR is detected (see below). The perikarya of serotonergic neurons are detected close to the anteriormost two commissures (sp, Fig. 11a). The processes extending from these cell bodies contribute to the major structures of the central nervous system, namely the prostomial neuropil, the respective commissures, the ventrolateral, paramedioventral and median nerve cords (Fig. 11a, b). Serotonin-LIR is also registered within the prostomial neuropil (np) and stomatogastric nerve ring (stnr, Fig. 11a). Additional, peripheral nerves are very poorly developed in the early stages and do not include strong serotonin LIR.

##### Hatching & early juvenile stages

*Central nervous system.* The parts of the nervous system shown by serotonin-LIR demonstrate significant development after hatching. The neuropil includes multiple serotonergic processes, whose perikarya are visible at the dorsoposterior part of the neuropil. Their processes seem to extend into the neuropil and these continue into both the dorsal and ventral root of the circumesophageal connective (Fig. 11c) and further on into the ventral nervous system.

The longitudinal cords of the ventral nervous system can be detected with serotonin-LIR, as being organized more loosely. The two anteriormost commissures are not strongly labelled and can therefore only be distinguished as very thin transverse nerve bundles or even solitary nerve fibres in the medium region of the body. All commissures are disguised by a loose plexus of nerves shown by serotonin-LIR (spl, Fig. 11d), but can be identified by perikarya shown with serotonin-LIR lateral to them along the entire ventral nervous system.

*Additional (dorsal, lateral, segmental) nerves.* Paired dorsolateral longitudinal nerves extend from the neuropil along the entire length of the body (lpn, Fig. 11c). Approximately five thin, intersegmental lateral nerves are labelled by the anti-serotonin antibody used. These nerves extend dorsally from the ventrolateral nerves towards the dorsolateral longitudinal nerves.

The stomatogastric nerve ring is very prominent and has an oval shape (stnr, Fig. 11c).

##### Adult

*Central nervous system.* Though the brain constitutes of 600–750 cells, serotonin-LIR can detect three pairs of them (data not shown). These cells are not clearly associated with specific organs or structures, although some of them are closer connected to the stomatogastric nerve ring and probably part of the stomatogastric nervous system. The proportion of the neuropil labelled with serotonin-LIR (snp, Fig. 11e) gives an even more uniform impression of the neuropil. Neither the dorsal nor the ventral root of the circumesophageal commissure can be clearly distinguished. Perikarya shown with serotonin-LIR are found lateral to the commissures in either the cellular accumulations in the anterior part of the ventral nervous system or as individual perikarya in the posterior region (sp, Fig. 11e). All cords of the ventral nervous system include fibres labelled by serotonin-LIR (mvn, pmvn, and vlnc, Fig. 11e). The commissures also show serotonin-LIR, whereby all three transverse nerve strands per segment in the anterior region and two in the two posterior segments can be recovered (acom, mcom, pcom 1–6, Fig. 11e).

*Additional (dorsal, lateral, segmental) nerves.* A plexus of nerve fibres is developed ventral to the ventral nervous system additional to the ventral nerve cords (spl, Fig. 11e). The pattern of the plexus is diffuse, but the plexus is only formed between the ventrolateral nerve cords (Fig. 11e). Serotonin-LIR can also be found in the transverse nerves emerging from the ventral nerve cords (ncb, nacb, npcb, nis, Fig. 11e), but is weaker for the longitudinal nerves, especially in the posterior part of the body, where both the dorsal and the lateral nerve fibres can hardly be seen.

*Stomatogastric nervous system.* Nerves form a ring around the esophagus (stnr, Fig. 11e) and are connected to the dorsal region of the neuropil, which is well represented by serotonin-LIR, though not as prominent as with acetylated α-tubulin-LIR (Fig. 6d, e). Several perikarya can be detected in younger adults dorsal to the region where these nerves emerge from the brain (spbr, Fig. 11e), but there is only a maximum of two perikarya shown by serotonin-LIR involved in the nerve ring around the pharynx (spst, Fig. 11e).

#### *Dinophilus gyrociliatus* dwarf male

Windoffer and Westheide [56] produced a reconstruction of the entire nervous system in high detail based on TEM-section-series. While 42 cells are reported to form the brain (which is approximately two thirds of the total amount of nerve cells in these animals [56]), cells shown by serotonin-LIR could only be detected in the posterior part of the body (Fig. 8b). They seem to be strongly connected to the penis, constituting the penis ganglia and the posterior proportion of the ventral ganglion. Thin nervous connections between these compounds could also be detected, most likely presenting the penis nerve cord (pnc, Fig. 8a). This cord connects the ventral ganglia to the penis ganglia. Additionally, some parts of the circumpenile nerve mass, which is embracing the penile sheath, could be revealed by serotonin-LIR. What appears to be the frontal gland and therefore in close association with the frontal ganglion was detected in the anterior region of the same specimen, consisting of a big cell detected with weak serotonin-LIR and unlabelled vesicles inside. Neither serotonin-LIR, nor FMRFamide-LIR could be detected in any immature developmental stages.

#### *Dinophilus taeniatus* (both sexes)

##### Embryonic development

The first neurons shown by serotonin-LIR differentiate at the level of the first commissure after several ciliary bands have been formed in *D. taeniatus*. Two pairs of perikarya are found lateral to the first formed commissures with projections into it (sp, Fig. 11f). Later on, more neurons labeled by serotonin-LIR are detected. In addition to the first neurons, the cells demonstrated by serotonin-LIR appear at the level of the second commissure. Their nervous processes extend into the second commissure and four additional perikarya are detected by serotonin-LIR at the dorsal part of the neuropil (spbr, Fig. 11g). Processes of these neurons extend through the neuropil into the dorsal and ventral roots, the circumesophageal connective, and then fuse with the serotonergic elements of the ventral nervous system, i.e. ventrolateral and median cords.

Late embryos of *D. taeniatus* add several structures revealed by serotonin-LIR, although the number of labeled perikarya does not increase significantly. Only a few additional cell bodies can be detected adjacent to the ventrolateral nerve cords; however, multiple nervous fibres are revealed by serotonin-LIR in the central nervous system as well as in peripheral nerves (Fig. 11h). In comparison to the previous stage, the components of the ventral nervous system labeled by serotonin-LIR spread posteriorly to the caudal tip of the embryo. The paramedioventral cords in the ventral nervous system include individual fibres showing serotonin-LIR similar to the ventrolateral and the median nerve cords (Fig. 11h, i). Serotonin-LIR supports the finding with acetylated α-tubulin LIR that the median nerve is constituted by two symmetrical nerves, which extend from the circumesophageal commissure and fuse at the level of the second commissure forming one median nerve (mvn). A thin connection is distinguished anterior to some commissures along the ventral nervous system, but not all (acom, Fig. 11h, i). Generally, the nerve fibres are not tightly condensed within nerve strands at this point in development. Both the stomatogastric nerve ring and four to five intersegmental lateral nerves are also detected by labeling with anti-serotonin antibodies before the animals hatch (Fig. 11h).

##### Hatching & early juvenile stages

*Central nervous system.* Nervous structures in the head do not change dramatically in juveniles of *D. taeniatus*. The neuropil with its roots includes a dense plexus shown by serotonin-LIR (np, Fig. 11j). The perikarya of four neurons revealed by serotonin-LIR are present in the dorsoposterior part of the neuropil (spbr, Fig. 11k). However, the ventral nervous system changes significantly: all cords get more condensed and prominent, so individual nerve fibres cannot be distinguished within them anymore. The transverse commissures are clearly labeled with serotonin-LIR. Remarkably, the thin transverse bundle anterior to the main commissure is not obvious in some segments, whereas traces are retained in mainly the anteriormost segments (acom, Fig. 11j). Paired groups of perikarya labeled by serotonin-LIR correspond to each main commissure, with their number varying between two and four perikarya in each ganglion-like accumulation. Ganglion-like structures corresponding to the first and second commissures consist of more compactly arranged cell bodies than those located more posterior. All longitudinal nerves of the ventral nervous system (ventrolateral, paramedioventral, and median nerve cords) converge towards the caudal part of the body and fuse in the posterior tip of a worm.

*Additional (dorsal, lateral, segmental) nerves.* The serotonin-LIR portion of peripheral nerves in juveniles shows several more additions than the hatchlings, with intersegmental lateral nerves extending laterally from the ventrolateral nerve cords (nis, Fig. 11k). These nerves are forming complete rings, while crossing the paired dorsoventral nerves and extending towards the unpaired dorsal nerve. These longitudinal nerves emerge from the dorsal part of the neuropil and extend to the caudal end of the body. Additional nerve fibres are randomly branching off from the major peripheral nerves.

The stomatogastric nerve ring is well represented by serotonin-LIR (stnr, Fig. 11j). It connects to the ventral part of the cerebral neuropil by a pair of short, but rather thick commissures. Two symmetrical groups, each consisting of two perikarya, can be detected near the lateral portion of the stomatogastric ring (spst, Fig. 11j).

##### Adult

*Central nervous system.* Generally, nerves revealed by serotonin-LIR constituting the central nervous system are packed very tightly therefore forming a precise pattern of nerve strands. The neuropil is a compact and dense nervous plexus in the brain (np, Fig. 11l). The ventral root of the neuropil is more prominent than the dorsal one. Both roots extend posteriorly and fuse at approximately the middle of the mouth opening to form the pair of circumesophageal commissures, which then fuse with the ventral nervous system. All nerves of the ventral nervous system include fibres shown by serotonin-LIR. The ventrolateral nerve cords are the most prominent components, while the paramedioventral and median nerve cords are rather thin. All longitudinal nerves can easily be distinguished since the processes revealed by serotonin-LIR constituting them are strongly condensed (Fig. 11l). This is unlike the state in *D. gyrociliatus*, where processes shown by serotonin-LIR constituting the main cords of the ventral nervous system are packed very loosely in adults (Fig. 11e). Therefore, the nervous system in *D. gyrociliatus* appears to be less defined as in *D. taeniatus*.

Although several transverse commissures in juveniles were supplemented by thinner, anterior nerve bundles, these additions are not retained in the adults, which therefore possess six prominent commissures separated by almost equal intervals except for the first two commissures (com1-6, Fig. 11l, m). These are closer together, which might be a result of the posterior displacement of the first commissure relative to the segment borders or the extension of the pharyngeal region. We therefore suggest that two bundles per segment found in prehatching stages condense during maturation. Several pairs of perikarya shown by serotonin-LIR are located lateral to each commissure, where they form ganglion-like structures. Thus, the ventral nervous system of the adult *D. taeniatus* is characterized by a regular organization, which is in stark contrast to the rather irregular distribution of commissures along the ventral nerve cord detected in adult *D. gyrociliatus* (Fig. 11e, l, m).

*Additional (dorsal, lateral, segmental) nerves.* The peripheral nerves of adults are very similar to those of juveniles, given the few other morphological differences between these stages. Four to five intersegmental nerves emerge perpendicular to the ventrolateral nerve cords and extend circumferentially around the body, forming dorsal connections between the two lateral cords (nis, Fig. 11m). Additional nerves innervate the ciliary bands, thereby tracing them along the body circumference (ncb, Fig. 11m). Dorsal and dorsolateral nerves extend longitudinally. Multiple individual fibres branching off from the lateral and longitudinal peripheral nerves create a loose intraepithelial network and supplement this grid-like system.

*Stomatogastric nervous system.* The only serotonin-LIR structure innervating the digestive system is the stomatogastric nerve ring (stnr, Fig. 11l). The second, posterior ring around the midgut-hindgut transition, which is also not labelled by acetylated α-tubulin-LIR, seems to be mainly labelled by FMRFamide-LIR in both species of *Dinophilus* (see below).

### Nervous components showing neurotransmitter-immunoreactivity: FMRFamide-LIR

#### *Dinophilus gyrociliatus* females

##### Embryonic development

**Fig. 12:**
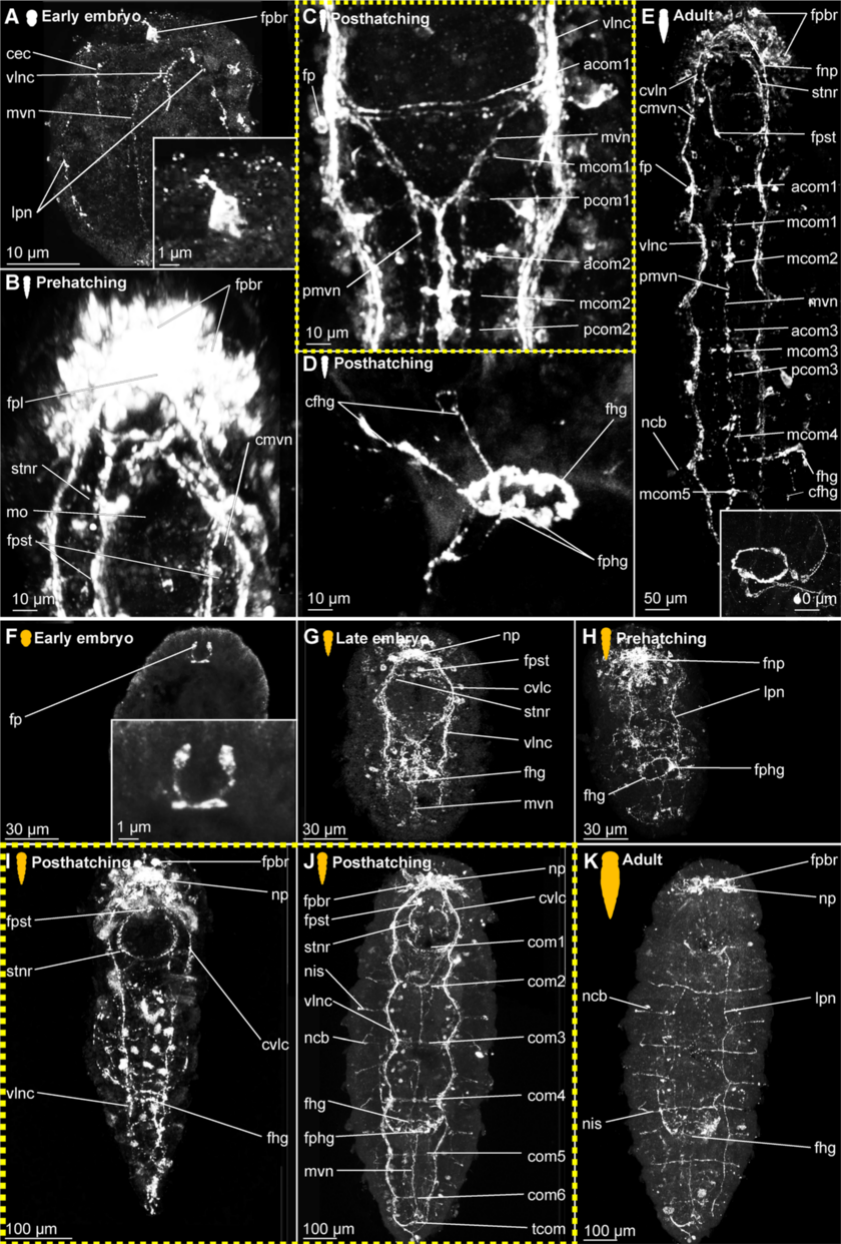
Neurogenesis in female *Dinophilus gyrociliatus* (**a**-**e**) and *D. taeniatus* (**f**-**k**) as revealed by FMRFamide-LIR. FMRFamide-like immunoreactive elements shown as white structures on black background, animals are oriented with the anterior end up. Stages are indicated by silhouettes next to the figure capture, and the assignment to the respective stage next to them. The first signs of difference between the two species *D. gyrociliatus* and *D. taeniatus* are emphasized by a yellow dashed-lined frame around the picture. **a** ventral view of the FMRFamide-like immunoreactive nervous system in early embryos of *D. gyrociliatus* with a detail of the first immunoreactive cell, **b** detail of the brain and anterior nervous system in a hatching female, **c** detail of the anterior trunk nervous system in an early juvenile female, **d** nerve ring around the midgut-hindgut-transition in a juvenile female, **e** ventral view of the FMRFamide-like immunoreactive nervous system in an adult female with detail of the nerve ring around the midgut-hindgut-transition, **f** ventral view of an embryo at ventral ciliary field stage of *D. taeniatus* with two immunoreactive cells with a process in the head region with a detail of these cells, **g** lateral view of a late embryo with multiple FMRFamide-like immunoreactive perikarya surrounding the neuropil and throughout the ventral nervous system, **h** dorsal view of a late embryo, **i** ventral view of a juvenile specimen **j** ventral view of an adult, **k** dorsal view of an adult. Abbreviations: acom – anterior commissure, cec – circumesophageal connective, cfhg – connection between the FMRFamide-like immunoreactive nervous ring around the midgut-hindgut-transition and the ventrolateral nerve cords, cmvn – circumesophageal commissure forming the medioventral nerve, com1-6 – commissure 1–6, cvln – circumesophageal commissure forming the ventrolateral nerve cord, fhg – FMRFamide-like immunoreactive ring around the midgut-hindgut transition, fp – FMRFamide-like immunoreactive perikaryon, fpbr – FMRFamide-like immunoreactive perikarya of the brain, fphg – FMRFamide-like immunoreactive perikaryon associated with the ring around the midgut-hindgut transition, fpl – FMRFamide-like immunoreactive plexus in the neuropil, fpst – FMRFamide-like immunoreactive perikaryon of the stomatogastric nerve ring, lpn – longitudinal peripheral nerve, mcom – median commissure, mo – mouth opening, mvn – medioventral nerve, nar – nerves innervating the anterior rim, pcom – posterior commissure, tcom – terminal commissure, vlnc – ventrolateral nerve cord

The first immunoreactivity to the anti-FMRFamide antibodies was revealed in a single cell during embryogenesis of *D. gyrociliatus*, when several ciliary bands are already formed. The perikaryon of this cell is found anterior to the neuropil and this cell produces several processes shown by FMRFamide-LIR, which extend towards the neuropil (fpbr, Fig. 12a). FMRFamide-LIR can also be detected within the median nerve as well as in the longitudinal peripheral nerves (mvn, lpn, Fig. 12a). During late embryogenesis, the processes of this neuron spread throughout the central nervous system and are detected in the neuropil and in ventrolateral nerves.

##### Hatching & early juvenile stages

*Central nervous system.* Juveniles of *D. gyrociliatus* demonstrate a much more complex pattern of FMRFamide-LIR in the nervous system. The neuropil consists of a dense plexus of immunopositive neurites. The neuropil is furthermore surrounded by multiple FMRFamide-LIR perikarya (fpbr, Fig. 12b). The processes showing FMRFamide-LIR extend from the brain into the circumesophageal connective and then into the ventrolateral and the median nerve cords. Paramedioventral cords and commissures also contain neurites labeled by FMRFamide-LIR (Fig. 12c).

*Additional (dorsal, lateral, segmental) nerves.* Nerve fibres shown by FMRFamide-LIR within the dorsolateral longitudinal nerves detected in early embryos are still clearly seen in juveniles. However, lateral nerves, which already develop at this stage, are not shown with FMRFamide-LIR.

The stomatogastric nerve ring is very prominent, with one pair of perikarya in the anterior region of the ring (fpst, Fig. 12b). The posterior ring around the midgut is less prominent, but also registered at this stage (fhg, Fig. 12d). A small number of perikarya (2–4) can be detected in the ring (fphg, Fig. 12d).

##### Adults

*Central nervous system.* The amount of elements shown by FMRFamide-LIR in the brain is higher than the number of serotonin-LIR-displaying perikarya, with a series of cell soma in closer association of the central neuropil (fpbr, Fig. 12e). They are connected to the central neuropil via short, thin nervous projections. They seem to be distributed equally dense laterally, anteriorly, and posteriorly to the neuropil, but there seem to be only few perikarya with this specific immunoreactivity seen ventral to the neuropil.

There are fewer nerve fibres shown with FMRFamide-LIR in the paramedian nerves than with serotonin-LIR (pmvn, Fig. 12e). However, several nerve fibres shown with FMRFamide-LIR are found in the ventrolateral longitudinal nerve cords (vlnc, Fig. 12e). The commissures along these bundles display a weak pattern, though at least the median transverse nerve strand can be clearly distinguished throughout the entire body length (mcom, Fig. 12e). Nevertheless, anterior neurite bundles (acom) are also detected by FMRFamide-LIR. Only a few perikarya expressing FMRFamide-LIR are found scarcely distributed along the ventral nerve cord (fp, Fig. 12e), in a lower number than the number of randomly distributed serotonin-LIR perikarya, but more densely arranged in the anterior part of the ventral nervous system.

*Additional (dorsal, lateral, segmental) nerves.* FMRFamide-LIR reveals less of the longitudinal and transverse nerves than serotonergic or acetylated α-tubulin-LIR, since only the transverse nerves underlying the ciliary bands (ncb, Fig. 12e), but not the longitudinal nerves can be detected.

*Stomatogastric nervous system.* Next to the ring around the pharynx emerging from the brain (stnr, Fig. 12e), which is very similar to the pattern depicted in serotonin-LIR besides including more perikarya (fpst, Fig. 12e), a second ring can be detected with FMRFamide-LIR in the posterior part of the body. It is formed around the constriction between mid- and hindgut (fhg, Fig. 12e) and seems to be constituted by one or two (closely adjacent) nerve rings, which are connected to the ventral nervous system.

#### *Dinophilus gyrociliatus* dwarf male

No immunoreactivity could be detected by using antibodies directed against FMRFamide-like peptides in the dwarf male (data not shown).

#### *Dinophilus taeniatus* (both sexes)

##### Embryonic development

The development of the nervous system components shown by FMRFamide-LIR begins with differentiation of two immunopositive cells at the anterior tip of an embryo. The perikarya of these cells are detected within the ectodermal sheet anterior to the neuropil, and their short processes project posterior into the neuropil (fp, Fig. 12f). As embryogenesis proceeds, additional FMRFamide-LIR neurons differentiate in the central nervous system. Multiple perikarya are scattered around the neuropil (fpbr, Fig. 12g, h). However, the two first perikarya can still be distinguished from the remaining ones at this stage due to their prominent location anterior to the neuropil. All cells shown by FMRFamide-LIR extend fibres into the neuropil and furthermore into the ventral nervous system. Additionally, several perikarya labeled by FMRFamide-LIR are scattered irregularly along the ventral nerve cords, in no specific arrangement vis à vis the commissures. Although FMRFamide-LIR is detected within the ventrolateral and median nerve cords, the loose organization of the nervous system in animals at this stage do not allow a clear characterization of elements in the paramedioventral nerve cords and commissures by FMRFamide-LIR (Fig. 12g).

In contrast to serotonin-LIR, FMRFamide-LIR is strongly represented within the digestive system, as is already slightly visible at this early stage. The stomatogastric nerve ring is constituted by processes extending from two perikarya located anterior to the pharynx (stnr, fpst, Fig. 12g), while another thin nerve ring embraces the midgut-hindgut transition (fhg, Fig. 12g, h).

The layout of the peripheral nerve net can be best detected in dorsal view with paired dorsolateral nerves emerging from the head neuropil and extending in caudal direction along the body (lpn, Fig. 12h). They are supplemented by five to six thin lateral nerves, which are perpendicular to the dorsolateral nerves and extend dorsally from the ventrolateral nerve cords.

##### Hatching & early juvenile stages

*Central nervous system.* After hatching, the elements of the nervous system revealed by FMRFamide-LIR become clearer as a result of the tighter arrangement of the nervous processes; loose irregular structures cannot be detected with FMRFamide-LIR anymore. The neuropil is compact, and multiple perikarya shown by FMRFamide-LIR surround it (fpbr, Fig. 12i). The two anterior perikarya already detected in previous stages are more prominent than others, suggesting that the first cells displaying FMRFamide-LIR differentiating during early neurogenesis retain their specific neurosecretory function. Only the ventral root of the circumesophageal connective demonstrates strong FMRFamide-LIR, while the dorsal roots are only weakly labelled. The ventrolateral nerve cords are the most prominent structure shown by FMRFamide-LIR in the ventral nervous system (vlnc, Fig. 12i). The cords are furthermore supplemented by several perikarya labelled by FMRFamide-LIR, which are scattered randomly along the ventral nervous system.

*Additional (dorsal, lateral, segmental) nerves.* The architecture of the lateral and dorsolateral nerves is similar to that of the embryos described above and does not change after hatching.

*Stomatogastric nervous system.* The digestive system demonstrates very well developed innervation provided by neurons positive for FMRFamide-LIR. The oval or circular stomatogastric nerve ring embraces the pharynx (stnr, Fig. 12i), and two perikarya displaying FMRFamide-LIR are located adjacent to the anterior region of the ring. Individual neurites organize a loose meshwork along the gut, with these fibres extending caudally and eventually fusing with the caudal nerve ring around the midgut-hindgut transition (fhg, Fig. 12i).

##### Adult

*Central nervous system.* The neuropil becomes more compact in the adults than in the juveniles of *D. taeniatus*, and the small perikarya of FMRFamide-LIR neurons accumulate adjacent to its lateral regions (Fig. 12j). Two big perikarya are found anterior to the dorsal region of the neuropil (fpbr, Fig. 12k). The processes of all brain cells revealed by FMRFamide-LIR project into the brain (whereby the dorsal root remains almost unlabeled), and into the epidermal cell layers situated anterior to the neuropil. However, individual fibres expressing FMRFamide-LIR are hard to distinguish within the neuropil. The signal in the ventral root of the circumesophageal connective, however, is very clear, and fibres extend caudally from the brain and can be traced within the ventral nervous system, which demonstrates a more regular organization in adult animals than in juveniles (Fig. 12j). The ventrolateral nerve cords shown by FMRFamide-LIR are the thickest bundles in the ventral nervous system, and multiple perikarya labeled by FMRFamide-LIR are scattered along their entire length. Evidently, their processes are involved in the formation of the latter. The median nerve cord contains two thin fibres converging at the level of the second commissure. In contrast to earlier stages, all six commissures can be depicted, and no additional nerve bundles anterior or posterior to the main commissures are visible (com1-6, Fig. 12j).

*Additional (dorsal, lateral, segmental) nerves.* In contrast to the serotonergic peripheral nerves, intersegmental nerves and nerves of ciliary bands labeled by FMRFamide-LIR have the same diameter so look similar at the dorsal view (nis, ncb, Fig. 12j, K). Dorsal nerves are also labeled with the anti-FMRFamide-like antibodies (lpn, Fig. 12k).

*Stomatogastric nervous system.* The stomatogastric nerve ring retains the organization established in the prehatching embryo with two perikarya shown by FMRFamide-LIR being located posterior to the neuropil and their projections embracing the pharynx, thus forming the stomatogastric nerve ring (stnr, Fig. 12j). Individual thin nerve fibres extend along the gut, forming a weak nerve net around it. The caudal nerve ring encircling the gut can be easily detected between the fourth and fifth neural commissures (fhg, Fig. 12j, k).

## Discussion

### Progenetic origin of female *D. gyrociliatus* from a juvenile* D. taeniatus*-like ancestor

Patterns in musculature, external ciliation and nervous system of adult female *D. gyrociliatus* all match the patterns found congruently in the late embryonic/early juvenile life stage of *D. taeniatus*.

**Fig. 13:**
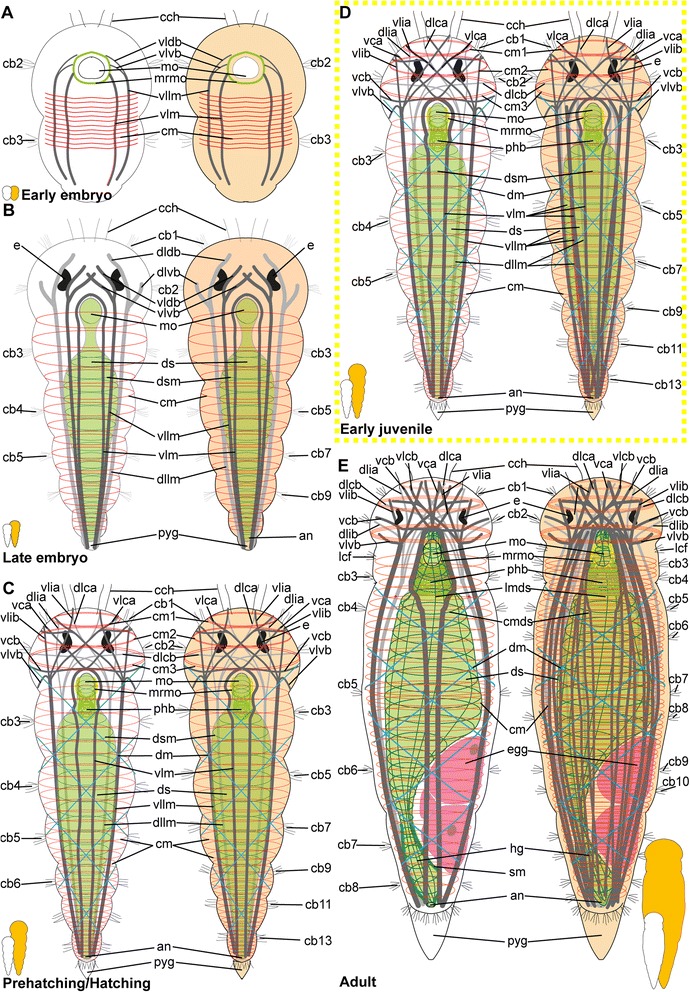
Comparative myogenesis in female *Dinophilus gyrociliatus* and *D. taeniatus*. The individual muscular groups are colour coded (circular muscles in red, ventral and ventrolateral longitudinal muscle bundles in dark grey, dorsolateral longitudinal muscle bundles in light grey, circular and longitudinal muscles of the digestive system in dark green); female *Dinophilus gyrociliatus* are depicted on the left hand, females of *D. taeniatus* on the right hand. Stages are indicated by silhouettes next to the figure capture (also indicating the size difference at this stage between the two species *D. gyrociliatus* in white and *D. taeniatus* in orange), and the assignment to the respective stage next to them. The first signs of difference between the two species are emphasized by a yellow dashed-lined frame around the picture. **a** Early embryo (3 days in *D. gyrociliatus* or 7–9 days in *D. taeniatus* after the eggs are deposited, respectively), **b** late embryo (5–5.5 or 12–14 days after the eggs are deposited, respectively), **c** prehatching females (5.5 or 13–20 days after the eggs have been deposited, respectively), **d** early juvenile, **e** adult females (older than 14 days or several weeks after the eggs are deposited, respectively). Abbreviations: an – anus, cb1-14 – ciliary band 1–14, cch – compound cilia of the head, cm – circular muscle, cmds – circular muscle of the digestive system, dlca – contralatero-anterior branch of the dorsolateral longitudinal muscle, dlcb – contralateral branch of the dorsolateral longitudinal muscle, dldb – dorsal branch of the dorsolateral longitudinal muscle, dlia – ipsilatero-anterior branch of the dorsolateral longitudinal muscle, dlib – ipsilateral branch of the dorsolateral longitudinal muscle, dlvb – ventral branch of the dorsolateral longitudinal muscle, dm – diagonal muscle, ds – digestive system, dsm – musculature of the digestive system, e – eye, egg – egg, hg – hindgut, lmds – longitudinal muscle of the digestive system, mo – mouth opening, mrmo – muscular ring around the mouth opening, phb – pharyngeal bulb, pyg – pygidium, vca – contralatero-anterior branch of the ventral longitudinal muscle, vcb – contralateral dorsal branch of the ventral longitudinal muscle, vlca – contralatero-anterior branch of the ventrolateral longitudinal muscle, vlcb – contralateral branch of the ventrolateral longitudinal muscle, vldb – contralatero dorsal branch of the ventrolateral longitudinal muscle, vldb – dorsal branch of the ventrolateral longitudinal muscle, vlia – ipsilaterial branch of the ventrolateral longitudinal muscle, vlib – ipsilatero-anterior branch of the ventrolateral longitudinal muscle, vllm – ventrolateral longitudinal muscle, vlm – ventral longitudinal muscle, vlvb – ventral branch of the ventrolateral longitudinal muscle

The onset of myogenesis, the addition of circular muscles, and the ramification that gives rise to the prostomial musculature are highly similar in the two investigated species (Figs. 2, 3, 5, 13). However, adult *D. taeniatus* possess four times the number of longitudinal body wall muscles than adult female *D. gyrociliatus* (Figs. 2h, 5e, g, h, 13)*,* whereas the prehatching stage of *D. taeniatus* presents a similar number to those of adult female *D. gyrociliatus* (three pairs of longitudinal muscles, Figs. 2f-h, 5d, 13).

**Fig. 14:**
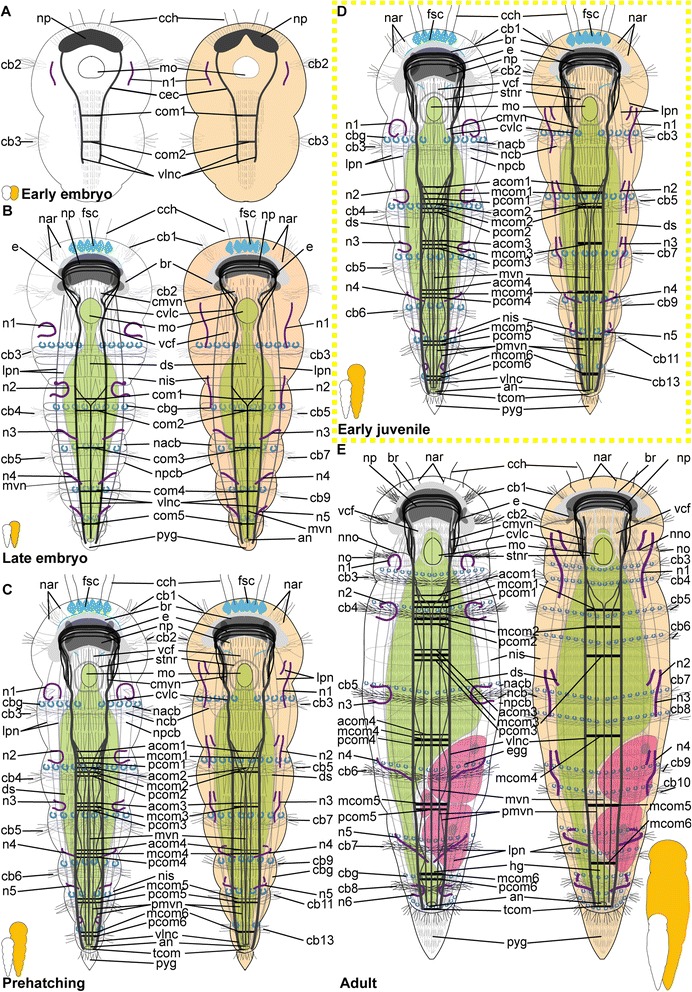
Comparative neurogenesis and development of ciliary patterns in *Dinophilus*. The acetylated α-tubulin-like immunoreactive structures are colour coded (ciliary bands and the ventral ciliary filed in light grey, the acetylated α-tubulin-like immunoreactive nervous system in dark grey, glandular structures (=pharyngeal glands, flask-shaped gland cells and ciliary band glands) in light blue, nephridia in dark purple); female *Dinophilus gyrociliatus* are depicted on the left hand, females of *D. taeniatus* on the right hand. Stages are indicated by silhouettes next to the figure capture (also indicating the size difference at this stage between the two species *D. gyrociliatus* in white and *D. taeniatus* in orange), and the assignment to the respective stage next to them. The first signs of difference between the two species are emphasized by a yellow dashed-lined frame around the picture. **a** Early embryo (3 days in *D. gyrociliatus* or 7–9 days in *D. taeniatus* after the eggs are deposited, respectively), **b** late embryo (5–5.5 or 12–14 days after the eggs are deposited, respectively), **c** prehatching females (5.5 or 13–20 days after the eggs have been deposited, respectively), **d** early juveniles, **e** adult females (older than 14 days or several weeks after the eggs are deposited, respectively). Abbreviations: acom – anterior commissure, an – anus, br – brain, cb1-14 – ciliary band 1–14, cbg – ciliary band gland, cch – compound cilia of the head, cec – circumesophageal connective, cmvn – circumesophageal connective forming the medioventral nerve, com 1–5 – commissure 1–5, cvlc – circumesophageal connective forming ventrolateral nerve cord, ds – digestive system, e – eye, fsc – flask-shaped cells, lcf – lateral ciliary field, lpn – longitudinal peripheral nerve, mcom – median commissure (= only commissure in *D. taeniatus* posthatching and adult stages), mo – mouth opening, mvn – medioventral nerve, n1-6 – nephridium 1–6, nacb – lateral nerve anterior to the ciliary band, nar – nerves innervating the anterior rim, nis – intersegmental lateral nerve, nlcf – nerve innervating the lateral ciliary field, np – neuropil, npcb – lateral nerve posterior to the ciliary band, pcom – posterior commissure, pmvn – paramedioventral nerve, pyg – pygidium, stnr – stomatogastric nerve ring, tcom – terminal commissure, vcf – ventral ciliary field, vlnc – ventrolateral nerve cord

The developmental patterns of external ciliation differ in the two investigated species. During embryogenesis, transverse ciliary bands and the ventral ciliary field emerge successively in *D. taeniatus* (ciliary band 2 (cb2) → ventral ciliary field (vcf) → cb1, cb3, 5, 7 → 9, 11, 13; Figs. 6, 9, 14), starting with the formation of one ciliary band per segment (cb1, cb3, 5, 7, 9, 11, 13) and during maturation adding an additional transverse ciliary band per body segment (cb4, 6, 8, 10, 12, 14) to create a total number of 14 ciliary bands. This differs from the developmental sequence observed in *D. gyrociliatus*, where the ciliary bands 1 and 3–7 originate simultaneous with the formation of the ventral ciliary field (cb2 → vcf, cb1, cb3-7, Figs. 6, 14) and an adult female *D. gyrociliatus* totally possesses eight ciliary bands (Figs. 6h, 14), equal to the number of ciliary bands observed in prehatching and juvenile *D. taeniatus* (Figs. 9e, 14).

**Fig. 15:**
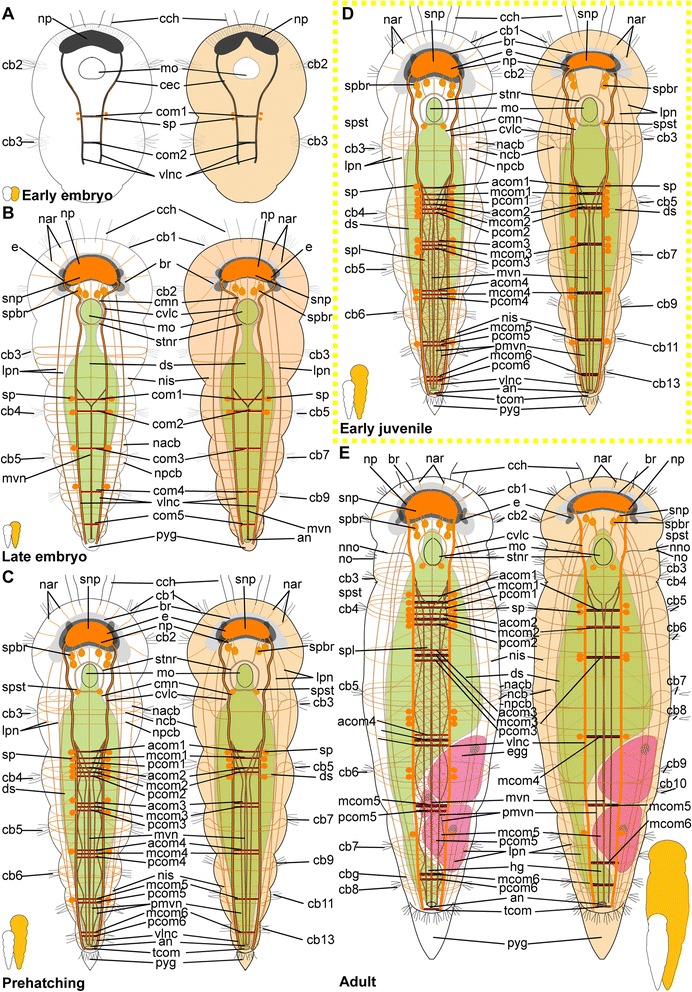
Comparison of the serotonergic nervous system in *Dinophilus*. The nervous structures are colour coded (acetylated α-tubulin-like immunoreactive structures are labelled in grey, serotonin-like immunoreactive elements in orange); female *Dinophilus gyrociliatus* are depicted on the left hand, females of *D. taeniatus* on the right hand. Stages are indicated by silhouettes next to the figure capture (also indicating the size difference at this stage between the two species *D. gyrociliatus* in white and *D. taeniatus* in orange), and the assignment to the respective stage next to them. The first signs of difference between the two species are emphasized by a yellow dashed-lined frame around the picture. **a** Early embryo (3 days in *D. gyrociliatus* or 7–9 days in *D. taeniatus* after the eggs are deposited, respectively), **b** late embryo (5–5.5 or 12–14 days after the eggs are deposited, respectively), **c** prehatching females (5.5 or 13–20 days after the eggs have been deposited, respectively), **d** early juveniles, **e** adult females (older than 14 days or several weeks after the eggs are deposited, respectively). Abbreviations: acom – anterior commissure, an – anus, br – brain, cb1-14 – ciliary band 1–14, cch – compound cilia of the head, cec – circumesophageal commissure, com1-5 – commissure 1–5, ds – digestive system, e – eye, lpn – longitudinal peripheral nerve, mcom – median commissure (= only commissure in *D. taeniatus* late hatchlings and adults), mo – mouth opening, mvn – medioventral nerve, nacb – lateral nerve anterior to the ciliary band, nar – nerves innervating the anterior rim, ncb – nerve of the ciliary band, nis – intersegmental lateral nerve, nlcf – nerve innervating the lateral ciliary field, np – neuropil, npcb – lateral nerve posterior to the ciliary band, pcom – posterior commissure, pmvn – paramedioventral nerve, pyg – pygidium, snp – serotonin-LIR share of the neuropil, sp – serotonin-LIR perikaryon, spbr – serotonin-LIR perikaryon of the brain, spl – serotonin-LIR plexus, spst – serotonin-LIR perikaryon of the stomatogastric nerve ring, stnr – stomatogastric nerve ring, tcom – terminal commissure, vlnc – ventrolateral nerve cord

**Fig. 16:**
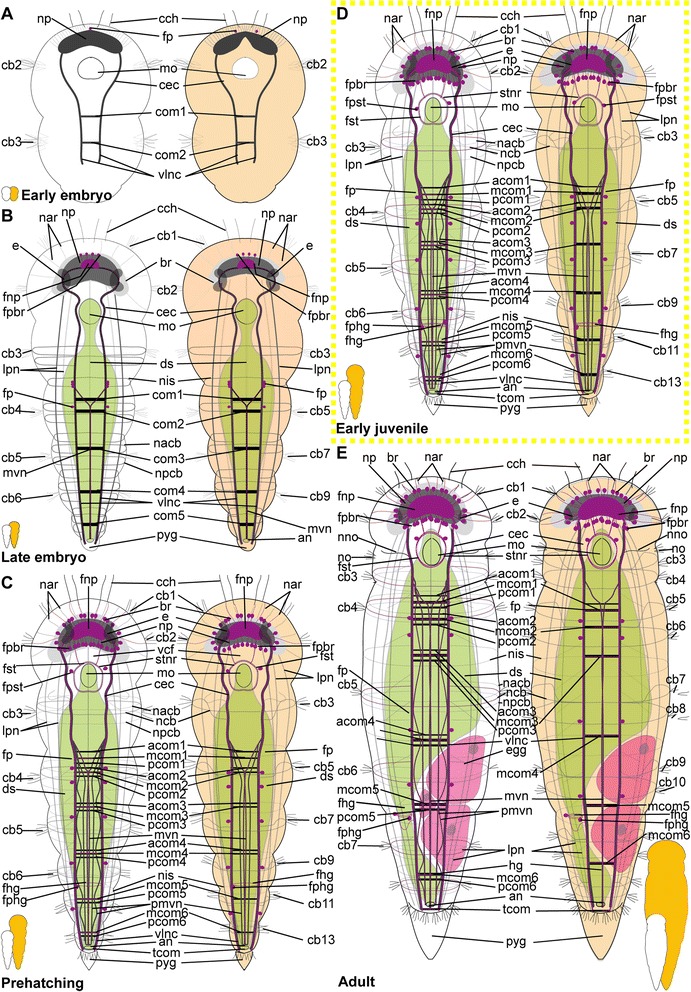
Comparison of the FMRFamide-like immunoreactive nervous system in *Dinophilus*. The nervous structures are colour coded (acetylated α-tubulin-like immunoreactive structures are labelled in grey, FMRFamide-like immunoreactive elements in dark purple); female *Dinophilus gyrociliatus* are depicted on the left hand, females of *D. taeniatus* on the right hand. Stages are indicated by silhouettes next to the figure capture (also indicating the size difference at this stage between the two species *D. gyrociliatus* in white and *D. taeniatus* in orange), and the assignment to the respective stage next to them. The first signs of difference between the two species are emphasized by a yellow dashed-lined frame around the picture. **a** Early embryo (3 days in *D. gyrociliatus* or 7–9 days in *D. taeniatus* after the eggs are deposited, respectively), **b** late embryo (5–5.5 or 12–14 days after the eggs are deposited, respectively), **c** prehatching females (5.5 or 13–20 days after the eggs have been deposited, respectively), **d** early juveniles, **e** adult females (older than 14 days or several weeks after the eggs are deposited, respectively). Abbreviations: acom – anterior commissure, an – anus, br – brain, cb1-14 – ciliary band 1–14, cbg – ciliary band gland, cch – compound cilia of the head, cec – circumesophageal commissure, com 1–5 – commissure 1–5, ds – digestive system, e – eye, fhg – FMRFamide-LIR ring around the midgut – hindgut transition, fnp – FMRFamide-LIR part of the neuropil, fp – FMRFamide-LIR perikaryon, fpbr – FMRFamide-LIR perikaryon of the brain, fphg – FMRFamide-LIR perikaryon of the nerve ring around the midgut-hindgut transition, fpst – FMRFamide-LIR perikaryon of the stomatogastric nerve ring, lpn – longitudinal peripheral nerve, mcom – median commissure (= only commissure in *D. taeniatus* late hatchlings and adults), mo – mouth opening, mvn – medioventral nerve, nacb – lateral nerve anterior to the ciliary band, ncb – nerve of the ciliary band, nar – nerves innervating the anterior rim, nis – intersegmental lateral nerve, nlcf – nerve innervating the lateral ciliary field, np – neuropil, npcb – lateral nerve posterior to the ciliary band, pcom – posterior commissure, pmvn – paramedioventral nerve, pyg – pygidium, stnr – stomatogastric nerve ring, tcom – terminal commissure, vlnc – ventrolateral nerve cord

Finally, during neurogenesis, one commissure is added per segment along the ventral nervous system in both species. Before hatching and in juveniles, this individual main commissure is supplemented by thinner, additional transverse neurite bundles in both species (Figs. 6d, g, 10c-e, 14, 15 and 16). After maturation these transverse bundles are condensed into one prominent, distinct commissural bundle in *D. taeniatus* (Figs. 11l, 12j, 14, 15 and 16), while female *D. gyrociliatus* retain a multi-commissural pattern into adulthood (three commissures in the anterior region of the ventral nervous system and two in the posterior area, also reported by Müller & Westheide [11]) (Figs. 6h, 11e, 12e, 14, 15 and 16). This addition (and in *D. taeniatus* later condensation) of nervous elements was observed with both pan-neural immunoreactivity (antibodies against acetylated α-tubulin) as well as with neurotransmitter markers (antibodies against serotonin and FMRFamide). Moreover, the adults of *D. taeniatus* demonstrate a more condensed configuration of nerve fibres and perikarya with serotonin-LIR and FMRF-amide-LIR than what is found in adult females of *D. gyrociliatus* (Figs. 11, 12, 15, 16); the latter hereby showing more resemblance to prehatching/juvenile *D. taeniatus*.

Heterochrony can be attested to each of these investigated organ systems, since in all patterns (musculature, nervous system, and ciliation) adult female *D. gyrociliatus* closely resembles the prehatching/early juvenile stage of *D. taeniatus*. This apparent congruence in change of timing leading to a simultanous arrest of development in three organ systems of the ancestor, renders strong support for a progenetic (as understood by [23]) origin of *D. gyrociliatus* from an ancestor with similar development as *D. taeniatus*.

As far as the organ systems investigated in this study are concerned, specific adult traits present in *D. taeniatus* were not found in adult *D. gyrociliatus*. This further supports that the latter evolved by a single speciation event rather than gradual miniaturization.

Without a reliable phylogeny of the family Dinophilidae it cannot be entirely ruled out that adult *D. taeniatus* could instead have originated by the respective overdevelopmental heterochronous process called hypermorphosis [19, 29]. However, this would demand that a late offset of somatic growth in three different organ systems of *D. taeniatus* resulted in both additions (as in the musculature and external ciliation) and condensations of elements (as in the nervous system) - which seems to be less likely. Additionally, the presence of dwarf males in *D. gyrociliatus* is likely a secondary specialization within Dinophilidae (with other dinophilids as well as most annelids having monomorphic sexes [83]), which supports a derived status of this species (through progenesis) rather than of *D. taeniatus*. Moreover, the prevalence of species within Dinophilidae resembling the morphology and often also the long life cycle of *D. taeniatus* [15, 32, 48–50, 53] does suggest a progenetic origin of *D. gyrociliatus* females from a *D. taeniatus*-like ancestor to be more likely than the alternative.

### Non-progenetic origin of dwarf male *D. gyrociliatus*

Whereas the overall dwarf male morphology did not resemble a specific life stage of the female *D. gyrociliatus,* some traits and developmental sequences are similar. The early developmental sequence of myogenesis in the dwarf males shows an onset of longitudinal muscles prior to development of circular muscle fibres in an anterior-to-posterior pattern and later addition of specialized muscular systems (Fig. 4), which resembles the pattern found in females of *D. gyrociliatus* as well as both sexes of *D. taeniatus*. In the adult dwarf males, comparatively few, and widely separated, longitudinal and circular muscles form the body wall musculature, whereas a dense grid of musculature surrounds the copulatory organ (Fig. 4 d). Interestingly, this musculature of the penile cone and its sheath show a similar layout to the much larger males of *D. taeniatus* (compare Figs. 4d-i, 5k). On the other hand, the presence of one pair of nephridia in the dwarf males possibly resembles the one pair found in early female embryos. The nervous system as well as ciliary system of the dwarf males is very limited and unique compared to the females. Their external ciliary structures are mainly found on the ventral side of the body, hence, not showing transverse ciliary rings (trochs), such as seen already during the early development in female *Dinophilus* or in the trochophore larvae of other annelids ([53], Fig. 8a, b). The similarity in dwarf male copulatory musculature to adult *D. taeniatus* copulatory musculature, co-occurring with ‘larval’ nephridia, as well as unique ciliation and somatic form, all in all contradicts that the dwarf males should have originated through a progenetic evolutionary process involving a somatic arrest in an early life stage of an ancestor. In the latter case the overall dwarf male morphology should instead in total resemble a specific early life stage of *D. gyrociliatus* or *D. taeniatus*- like ancestor, and not a mix of juvenile-, adult male- and unique characters. The current scenario instead suggests that more complex mechanisms underlie the evolution of dwarf males, possibly involving both various heterochronous events as well unique adaptations.

## Conclusions

The layout of musculature, nervous system and ciliation in adult female *D. gyrociliatus* was shown in this morphological study to closely resemble the prehatching/early juvenile rather than the adult layout in *D. taeniatus*. This global similarity to a younger life stage indicates a paedomorphic origin of female *D. gyrociliatus* from a *D. taeniatus*-like ancestor. Of the various heterochronous processes resulting in underdevelopment, progenesis is defined as an early offset (=arrest) of somatic development due to early maturation [10, 14, 23], which would have caused a simultaneous (global) change in development of several organ systems. We therefore suggest that a single speciation event (in the form of progenesis) rather than unrelated evolutionary changes took place in the formation of *D. gyrociliatus.*

The mechanisms underlying the development of the dwarf male could not be clarified yet, since its adult body plan shows reductions (in e.g. complexity of organ systems, cell number and external features) that does not congruently reflect a specific developmental life stage of the female *D. gyrociliatus*.

These evolutionary conclusions are based on the detailed atlas of the development of ciliary structures, musculature and nervous system in a group of direct developing meiofaunal annelids. This detailed morphological atlas therefore is one of the first done for direct developing meiofauna and may serve as a comparative basis for broader evolutionary studies, as well as a map onto which genetic patterning of one of these organ systems is allowed for.
